# TinderMIX: Time-dose integrated modelling of toxicogenomics data

**DOI:** 10.1093/gigascience/giaa055

**Published:** 2020-05-25

**Authors:** Angela Serra, Michele Fratello, Giusy del Giudice, Laura Aliisa Saarimäki, Michelangelo Paci, Antonio Federico, Dario Greco

**Affiliations:** 1 Faculty of Medicine and Health Technology, Tampere University, Arvo Ylpön katu 34, 33520, Tampere, Finland; 2 BioMediTech Institute, Tampere University, Arvo Ylpön katu 34, 33520, Tampere, Finland; 3 Institute of Biotechnology, University of Helsinki, Viikinkaari 5, 00014, Helsinki, Finland

**Keywords:** toxicogenomics, gene expression, integrated modeling, dose-response, time course, BMD, dynamic dose-dependent, mechanism of action, MOA

## Abstract

**Background:**

Omics technologies have been widely applied in toxicology studies to investigate the effects of different substances on exposed biological systems. A classical toxicogenomic study consists in testing the effects of a compound at different dose levels and different time points. The main challenge consists in identifying the gene alteration patterns that are correlated to doses and time points. The majority of existing methods for toxicogenomics data analysis allow the study of the molecular alteration after the exposure (or treatment) at each time point individually. However, this kind of analysis cannot identify dynamic (time-dependent) events of dose responsiveness.

**Results:**

We propose TinderMIX, an approach that simultaneously models the effects of time and dose on the transcriptome to investigate the course of molecular alterations exerted in response to the exposure. Starting from gene log fold-change, TinderMIX fits different integrated time and dose models to each gene, selects the optimal one, and computes its time and dose effect map; then a user-selected threshold is applied to identify the responsive area on each map and verify whether the gene shows a dynamic (time-dependent) and dose-dependent response; eventually, responsive genes are labelled according to the integrated time and dose point of departure.

**Conclusions:**

To showcase the TinderMIX method, we analysed 2 drugs from the Open TG-GATEs dataset, namely, cyclosporin A and thioacetamide. We first identified the dynamic dose-dependent mechanism of action of each drug and compared them. Our analysis highlights that different time- and dose-integrated point of departure recapitulates the toxicity potential of the compounds as well as their dynamic dose-dependent mechanism of action.

## Introduction

Toxicogenomic methods are widely used for the assessment of chemical hazards and environmental health [1]. Omics technologies have been broadly accepted and recognized as efficient and reproducible tools to study the effects of chemical exposures on different organisms [2]. In particular, transcriptomics technologies allow the investigation of gene expression patterns of thousands of genes after exposure to a substance [3] and they have been widely used in toxicogenomics [4–7].

Direct effects of chemical insults are generally expected to follow a monotonic dose-response alteration resulting in increasing effect as the dose increases until a plateau is reached [8]. In classical toxicology, this is observed as an increasing number of deceased animals or any other measurable apical end point. A similar effect can be appreciated in gene expression alteration. Because the changes in gene expression occur much earlier than apical end points, transcriptomic data can be used as a complementary approach to estimate relevant points of departure (PODs) and hence provide valuable information for the toxicological assessment [9, 10].

Until now, the no-observed-adverse-effect-level [11] and the benchmark dose (BMD) [12] methods have been used to help the interpretation of transcriptomic dose-response data and to derive transcriptomic PODs. In particular, by modelling the patterns of dose-responsiveness of gene expression, the BMD method provides an estimate of the lowest dose of a chemical able to induce a significant change in biological activity.

However, assaying the mechanism of action (MOA) of exposure at multiple time points is valuable to highlight useful information on the kinetic molecular responses of a biological system. For example, the experimental set-up for the *in vivo* studies in the Open TG-GATEs involves animal treatment in which each drug is tested at 3 doses (low, medium, and high) at multiple time points [7]. Thus, 1 major challenge is the interpretation of these toxicogenomics data, especially for identifying patterns of alterations of the gene expression that are correlated to both the dose levels and the time points. Different methods have been proposed to identify genes that show a dose-response effect [13,14] or to study the gene expression dynamics at individual time points [15,16]. However, this kind of approach does not allow an easy interpretation of the time-dependent dynamics happening after the exposure and the molecular adaptation process. To date, few efforts have been done to propose a methodology able to identify sets of genes that show similar expression patterns with respect to both dose and time [17].

In the present work, we propose a new computational framework, TinderMIX, for dose- and time-dependent gene expression analysis that aims to combine dimensionality reduction, BMD analysis, and polynomial fitting to find groups of genes that show a dynamic dose-response (DDR) behaviour. The integrated modelling used in TinderMIX allows us to interpolate the continuous joint dose-time space and predict the molecular alteration values for the doses and time points not included in the original experiment. Moreover, our approach is importantly able to inform on POD in both the dimensions of the doses and time points, hence resolving at once 2 analytical tasks that, thus far, have only been carried out subsequently to each other.

To illustrate our methodology we analysed the gene expression data for cyclosporine A and thioacetamide from the Open TG-GATEs database.

## Materials and Methods

**Figure 1:**
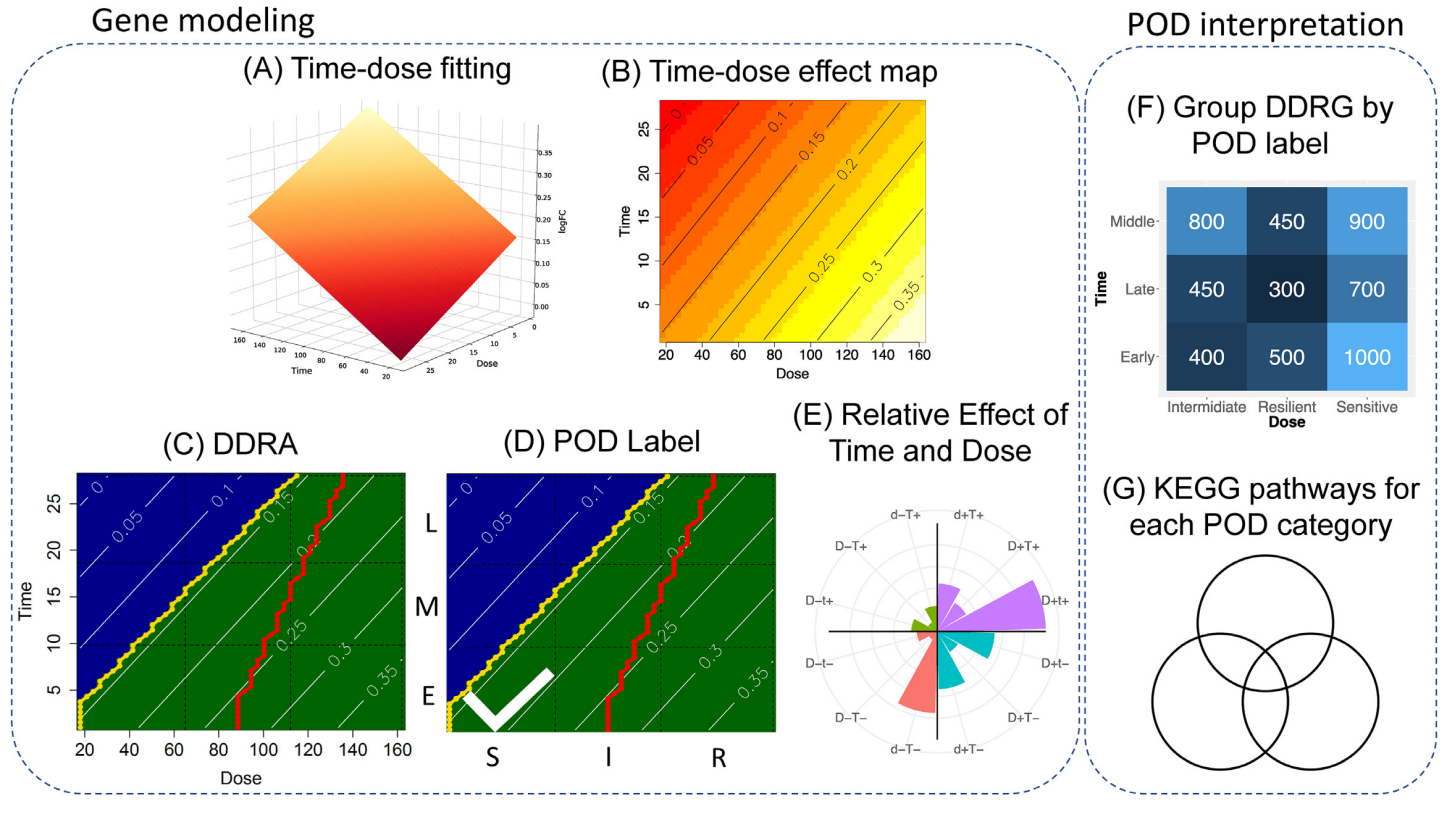
TinderMIX methodology. The TinderMIX methodology is composed of several steps that are grouped into gene modelling and POD interpretation. First, starting from the pairwise log fold-change of each gene a 3D polynomial model is fitted (A). Second, the 3D model is mapped in its 2D effect map by means of contour plots. The lines in the contour plots describe the shape of the 3D model by showing the portion of the space where the fitted model has a constant log fold-change, indicated by the value on the lines. A positive value means that the gene is upregulated; otherwise it is downregulated (B). Third, the dynamic dose-responsive area (DDRA) is identified. Starting from a user-defined activity threshold, the time-dose effect map is divided into regions of different colors. A blue region is a part of the map where the log fold-change is lower than the threshold, thus marked as a non-active area. A green or red area indicates an activation, with a log fold-change greater than the threshold. In particular, a green area (called “dynamic dose-responsive increasing area”) is characterized by an increase of the log fold-change when the dose increases, while a red area (called “dynamic dose-responsive decreasing area”) presents a decrease of log fold-change when the dose increases. In the example shown in (D) the highlighted area is colored in green, indicating an increasing log fold-change with respect to dose. A gene is considered DDR if there is a DDRA in the activity map, with a monotonic behaviour of the log fold-change from a dose to the highest one tested. In the map, the front of the dose-responsive area is marked with a yellow line. Moreover, a red line is used to mark the IC50 front, which is the line connecting the set of doses (1 for each time point) that specifies the 50% of gene activity (C). Fourth, based on the first dose and time of activation the gene receives an activation label that specifies its dose and time POD. The time-dose effect map is divided into a 3 by 3 grid. The sections of the dose axis are named “Sensitive” (S), “Intermediate” (I), and “Resilient" (R), while for the time axis the labels “Early” (E), “Middle” (M), and “Late” (L) are assigned. The final label was then obtained by identifying the earliest and most sensitive point of activation and concatenating the dose and time of the single labels (D). Fifth, the effect of the dose and time in the DDRA is compared (E). The second part of the analysis consists of the POD interpretation. The letters “d” and “t” stand for dose and time, respectively. In the label, a capital letter means that there is a stronger effect of a variable with respect to the other. If the 2 letters are both capitals, it means that the effect is of similar intensity. Concordance in the increase of the fold-change and dose/time is indicated with a plus sign, and decrease with a minus sign. Thus, the DDRGs identified are grouped by their POD labels (F). The colors in (F) indicate the amount of DDRGs for each POD, ranging from lower numbers depicted in dark blue, to higher numbers depicted in light blue. Eventually a pathway enrichment analysis for the genes in each category is performed (G).

The TinderMIX methodology proposed in this study starts from the sample-wise log fold-change of the genes and is able to identify which of them show a DDR behaviour and to estimate their joint dose-time POD. The methodology is composed of multiple steps that can be grouped into 2 parts: the gene modelling with POD identification (Fig. 1) that is executed for every gene in the dataset, and the POD interpretation of the dynamic dose-responsive genes (DDRG) identified in the first part (Fig. 1).

As for the classical BMD analysis, the first step of the gene modelling analysis (Fig. 1A) consists of fitting different polynomial functions to each gene log fold-change and identifying the optimal one by using a nested model hypothesis test [18]. The difference with the classical BMD analysis performed so far is that the fitted models are 3D functions in the space of the dose, log fold-change, and time. Indeed, so far, the BMD analysis has mainly been performed in the dose and log fold-change space for each time point individually. Afterward, the optimal 3D model is mapped in the dose-time dimensional space by means of contour plots (Fig. 1B). A monotonically increasing or decreasing (with respect to the doses) dynamic dose-responsive area (DDRA) is identified starting from a user-specified activation threshold. (Fig. 1C). If this DDRA is present, the gene is considered DDR and its POD is identified on the basis of the first dose level and activation time (Fig. 1D). Moreover, the effect of dose and time in the DDRA is compared to elucidate whether the modifications in the log fold-change values are due to a positive or negative effect of the time and dose and also to elucidate which of the 2 variables contribute most (Fig. 1E). Indeed, if the time has a stronger effect compared to the dose we can hypothesize that the effect under analysis can be an adaptation process; on the other hand, if the dose contributes the most we can hypothesize that we are observing an effect directly correlated to the exposure. In the second part of the analysis, the DDR genes identified can be grouped according to their POD labels (Fig. 1F). Eventually, overrepresented pathways can be calculated for each gene category (Fig. 1G).

### Time-dose fitting

For every gene, linear regression models including first-, second-, and third-order polynomials (Eqs 1–3) of the explanatory variables (log fold-change) are fitted. These models are selected as representative of those used in classical BMD analysis [8,12, 13]. Indeed, unlike the γ and logistic models, polynomials are easily generalized to >1 input variable and they can be used for reliable approximation of other functions.

(1) $$\begin{eqnarray*}
\mathrm{LFC} &=& \beta _{0} + \beta _{1}\mathrm{Dose} + \beta _{2}\mathrm{Time}
\end{eqnarray*}$$

(2) \begin{eqnarray*} \mathrm{LFC} &=& \beta _{0} + \beta _{1}\mathrm{Dose}^2 + \beta _{2}\mathrm{Time}^2\nonumber \\ && + \beta _{3}(\mathrm{Dose} \times \mathrm{Time}) + \beta _{4}\mathrm{Dose} + \beta _{5}\mathrm{Time} \end{eqnarray*}

(3) \begin{eqnarray*} \mathrm{LFC} &=& \beta _{0} + \beta _{1}\mathrm{Dose}^3 + \beta _{2}\left(\mathrm{Dose}^2 \times \mathrm{Time}\right)\nonumber \\ && + \, \beta _{3}\left(\mathrm{Dose} \times \mathrm{Time}^2\right) + \beta _{4}\mathrm{Time}^3 + \beta _{5}\mathrm{Dose}^2\nonumber \\ && + \, \beta _{6}\mathrm{Time}^2 + \beta _{7}(\mathrm{Dose} \times \mathrm{Time}) + \beta _{8}\mathrm{Dose} + \beta _{9}\mathrm{Time} \end{eqnarray*}

The fitting was performed by using the R lm function from the stats package [19]. The best-fitting model is selected performing a nested model hypothesis test [18]. The test is performed using the R function analysis of variance (ANOVA), which takes a list of models as an input. Each model in the list is considered as the “full” model with respect to the previous “restricted” model in the list, where a number of partial regression coefficients are set to 0. In other words, a model is restricted, with respect to the full model, in the sense that it does not consider 1 or more explanatory variables. For each pair of subsequent models in the list, we test the significance of the amount of variance explained by a subset of predictors of the restricted model, compared to the variance explained by all the variables of the full model. We also add to the test, at the head of the list of models, an implicit constant model in which all the partial regression coefficients are 0, except for the intercept β_0_. This is done to assess the quality of the fit in the first place. If the *P*-values corresponding to testing the models in Eqs 1–3 against the constant model are not significant, that gene is not considered as having a dose-response effect. If, on the other hand, there is a significant amount of variance explained by any of the models in Eqs 1–3, then we return the fitted model corresponding to the lowest *P*-value.

### Time-dose effect map prediction and dynamic dose-responsive gene identification

For each gene, the selected model is used to predict an activity map with the values of a smooth log fold-change function on a grid of 50 × 50 points covering the entire range of doses and time points tested. This map is represented as a contour plot and used to identify the dose-response area. A desired activity threshold corresponding to a 10% increase/decrease with respect to controls is set to identify the responsive area of each gene time-dose effect map. If a gene does not show an activity satisfying the threshold, it is removed from the analysis. A gene is also removed from the analysis if the responsive area does not include the highest dose of the experimental setting. The selected threshold of 10% is a default threshold used in BMD analysis of transcriptomics data [8,12, 13]. For the genes passing the previous filtering, the gradient of the smooth log fold-change function is computed at each point of the 50 × 50 grid. Each point of the grid is segmented according to whether its connected components show a monotonically varying (with respect to the whole dose range) increment or decrement of the log fold-change values. If only 1 component, with either increasing or decreasing behaviour, is present in the map and it contains the highest dose, then the component is selected as the candidate DDRA. However, if the highest dose is not contained in the area, then the gene is considered not responding and removed from the analysis. On the other hand, if multiple components with different increasing or decreasing behaviour are present in the map, 1 of the 2 is selected as candidate dose-responsive area, according to the criteria described in Additional File 1. In particular, each candidate region is weighted according to the following criteria:

(4) $$\begin{eqnarray*}
r_i &=& n_p + n_{\mathrm{tp-md}} - m_d, i \in \lbrace r_1, \ldots , r_n \rbrace,
\end{eqnarray*}$$

where *n_p_* is the number of points included in the region, *n*_tp-md_ is the number of time points (rows in the gene map) that are included in the candidate region that include the highest dose, *m_d_* is the minimum dose covered by the candidate region, and *n* is the number of candidate regions found in the gene maps. The optimal region is selected as the one maximizing the score. The active region is further reduced, by removing time points that are still active (log fold-change > 1.1) outside the optimal region but with a non-monotonic behaviour compared to the one inside the optimal region.

We identify the external borders of the segmented responsive area using a trace-boundary algorithm. The dose-responsive front is identified as the smallest dose present in the responsive area for each time point. Moreover, the IC50 front is also computed as the doses that give 50% of changes in the log fold-change at every time point.

### POD label assignment

**Figure 2:**
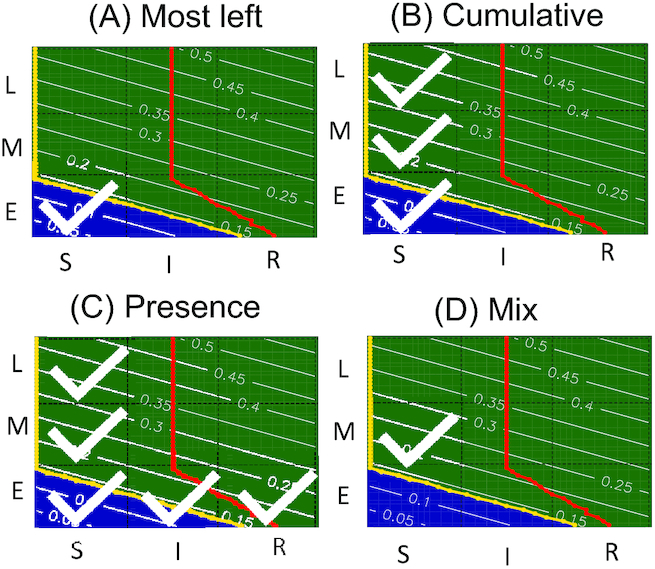
Different methodologies implemented to identify the dynamic dose-response POD on a sample gene map. With the “most left” method (A) the POD area is marked as sensitive-early because the activation DDRA is already present at the lowest dose and shortest time point. With the “cumulative” method (B), the POD is marked as sensitive-early, sensitive-middle, and sensitive-late. The label is obtained by using 80% of the cumulative length of the DDRA border (yellow line). With the “presence” method (C) the POD is labelled as sensitive-early, sensitive-middle, sensitive-late, intermediate-early, and resilient-early. With the “mix” method (D) the POD is labelled as sensitive-middle because the DDRA starts already at lower doses and shorter time points and the area is also the one with a higher coverage by the DDRA.

The dose-time space is partitioned into 3 × 3 regions, where each dose can be labelled as sensitive, intermediate, or resilient and each time point is labelled as early, middle, or late. Different strategies to identify the POD of the DDRGs are implemented in TinderMix (Fig. 2). The first method is called the “most left” strategy (Fig. 2A). It looks for the first available POD region by identifying the most sensitive point of activation (lowest dose) and then its corresponding earliest time point. The second method, called “cumulative,” ranks the regions in the 3 × 3 scheme based on the percentage of points of the border of the DDRA that they contain. Then the regions needed to cover the X% of the border are identified and marked as POD (Fig. 2B). X is a threshold specified by the user. In case of X = 100, the “cumulative” method gives the same result as the “presence” method. The third strategy, called “presence,” identifies as PODs all the regions containing the DDRA border marked as a yellow line (Fig. 2C). The fourth method, called “mix,” creates a score for each region that takes into account how close the region is to the lowest dose and earliest time point, how much area of the region is covered by the DDRA, and how many points of the DDRA border are contained in it. The region with the higher score is selected as POD (Fig. 2D). When the most sensitive and most early region is also the one with higher coverage, the mix and most left methods give the same results. In all the approaches the final POD label is obtained by concatenating the dose and time of the single labels. For example, a gene with label sensitive-early is a gene that shows a response already at low doses and early time points. On the other hand, a resilient-late gene shows a response only at high doses and late time points. It is important to highlight that the labelling strategies are meant to give an indication of the POD, but they are not self-explanatory of the whole gene expression dynamic. The presence and cumulative approaches identify multiple regions as POD, giving a better approximation of the shape of the DDRA area. These 2 approaches are suggested when the focus of the study is on a few genes. On the other hand, the most left and mix approaches identify only the starting point of the gene activation and can be easily used to group genes and give biological interpretation of a chain of events. In the analyses conducted in this work we have used the most left approach because it follows the toxicological assumption that a toxicant is considered active at the lowest dose and earliest time point at which its effect is significantly deviating from the control status.

### Time and dose influence by means of gradient

Once the DDRA is identified, the influence of time on the fold-change is studied by analysing the vector field of gradients in the region. For each point, the magnitude and the angle of the gradient are computed and used to evaluate the time-dose response score as follows:

(5) $$\begin{eqnarray*}
td_s &=& \frac{1}{\sum _{i=1}^n \mathrm{mod}_i} \times \left(\sum\nolimits _{i=1}^n g_i \times \mathrm{mod}_i\right),
\end{eqnarray*}$$

where *g_i_* and mod*_i_* are the angle and magnitude of the gradient in the *i*-th point in the DDRA and *n* is the number of points in the same region. This score can be used to categorize the genes on the basis of the dose and time effect on their fold-change. According to the time-dose response score, the active genes are divided into 4 groups, corresponding to the 4 quadrants of Cartesian space. In the first quadrant the fold-change increases with both time and dose (0 < *td_s_* ≤ 90); in the second quadrant the fold-change increases with time but decreases with dose (90 < *td_s_* ≤ 180); in the third quadrant the fold-change decreases with both time and dose (180 < *td_s_* ≤ 270); in the fourth quadrant the fold-change increases with dose and decreases with time (270 < *td_s_* ≤ 360). The 4 quadrants can be further dissected in 3 smaller sectors: 1 in which the dose has a stronger effect than the time, 1 in which they have the same effect, and 1 in which the time has a stronger effect than the dose. We assign a label to each gene composed by the letters *d* and *t*,standing for dose and time, respectively, and a positive or negative sign. If the influence of 1 of the 2 components is stronger than the other, this is highlighted by using capital letters. For example, the label *d* + *T* + stands for fold-change increasing with both dose and time, with a stronger effect from the time.

### Enrichment of biological pathways

KEGG enrichment analysis is performed by using the methodology implemented in the FunMappOne tool [20]. Pathways were considered significantly enriched if they have a corrected *P*-value < 0.05.

### Dataset collection

Pathological events registered in rats after drug exposure were downloaded from the Open TG-GATEs database [7, 21]. Every drug pathological score was computed by counting the number of events occurring after the exposure normalized by the number of events in the controls. Cyclosporine A and thioacetamide were selected as representative candidates for drugs with low and high toxic impact, respectively [22–25].

Raw data microarray transcriptomic data for cyclosporine A and thioacetamide exposure, in *in vivo* rat liver tissue, were downloaded from the Open TG-GATEs dataset [7, 21]. Each dataset consists of 48 samples, of which 12 are unexposed controls and 12 are biological replicates for each dose level. Cyclosporine A was tested with doses 30, 100, and 300 mg/kg, and thioacetamide, with doses of 4, 15, and 45 mg/kg. In both cases, samples were harvested at 4 different time points (3, 6, 9, and 24 hours). Transcriptomics data were preprocessed using the pipeline implemented in the eUTOPIA tool [26]. The raw data were imported into R v. 3.4 by using the justRMA function from the Bioconductor utilities [27] to annotate probes to Ensembl genes (by using the rat2302rnensgcdf [v. 22.0.0] annotation file from the brainarray website [28]) and quantile normalize the resulting expression matrix. Next, the experimental batch effects due to technical variables were estimated and removed using the ComBat algorithm implemented in the Sva package [27,29]. For each pair of dose and time point, all the pairwise log fold-changes for each gene were computed as the difference between the log_2_ expression values of each pair of treated and control samples. In this way, we obtained 108 pair-samples and 11,721 genes to be used in the TinderMIX analysis.

## Results and Discussion

We developed a novel dose and time integrative modelling strategy for transcriptomics data able to identify molecular features with a DDR alteration pattern (Fig. 1). We showcase our methodology by analysing *in vivo* gene expression data for 2 drugs (cyclosporine A and thioacetamide) available in the Open TG-GATEs dataset.

### Dynamic dose-dependent MOA

The TinderMIX methodology allows dy th ofe distribution of the gene log fold-change with respect to both dose and time. For this purpose, TinderMIX implements a strategy similar to the classical BMD analysis [12] but translated into a 3D space.

For every gene, linear and second- and third-order polynomial models are fitted (Fig. 1A). The optimal model is selected as the one with adjusted goodness-of-fit *P*-value < 0.01 and best modelling performance according to the nested model hypothesis test, as performed by ANOVA. Furthermore, for the genes that pass the goodness-of-fit filtering, their dynamic dose-responsiveness is investigated. Hence, TinderMIX maps the 3D optimal model in its corresponding time-dose effect map by means of contour plots (Fig. 1B). This step allows the DDRA to be identified, by iterating the concept of dose-responsiveness on each time point (Fig. 1C). We consider a gene to be dose-dependently altered if its response is monotonic throughout all the doses at any time point. Given the complex kinetics of gene expression, interpolating the behaviour of the genes between the tested doses increases the robustness of dose-response modelling when multiple time points are assayed.

Starting from a standard gene activation threshold of 10%, TinderMIX identified 5,746 and 8,436 dose-responsive genes in cyclosporine A and thioacetamide, respectively (Table 1). In the case of cyclosporine A, most of the genes were fitted by the second-order polynomial model, while for thioacetamide by the third-order polynomial model (Table 1). This suggests that there is a predominant non-linear relationship between the log fold-changes, the dose levels, and the time points. The complete lists of DDR genes for cyclosporine A and thioacetamide are available in Additional Files 2 and 3, respectively.

**Table 1.** The number of dynamic dose-responsive genes

Drug	DDRGs	Linear	Poly2	Poly3
Cyclosporine A	5,746	1,362	4,164	220
Thioacetamide	8,436	2,352	1,031	5,053

DDRG: dynamic dose-responsive gene. Linear, Poly2, and Poly3 are the numbers of DDRGs that are fitted by a linear, second-, and third-order polynomial function, respectively.

Furthermore, the sensitivity of the TinderMIX method to the activation threshold was investigated. The analyses were run for different activation thresholds (10%, 20%, 30%, 40%, and 50%). In both drugs in our case study, the number of DDRGs decreases with increasing threshold (Additional File 4).

### TinderMIX labelling for point of departure

**Figure 3:**
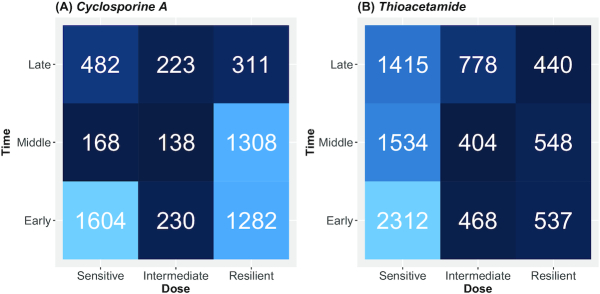
The number of dose-responsive genes for each gene category in cyclosporine A (A) and thioacetamide (B). The colors indicate the amount of dose-responsive genes for each gene category, ranging from lower numbers depicted in dark blue, to higher numbers depicted in light blue.

By identifying the first dose and time point present in the DDRA (Fig. 1C), TinderMIX assigns to each DDRG an activity label (Fig. 1D). The label provides information on the joint time-dose POD at a glance. For instance, the labels aid drawing a hypothetical sequence of events. The fact that a gene can respond at different dose ranges informs on the sensitivity of certain molecules and regulatory machinery to a specific exposure. The analysis of the DDRG profiles might give insights about the harmfulness of a compound. Indeed, substances that activate many genes at low doses are probably more toxic than those exerting resilient responses. This is the case of the 2 drugs we analysed, as cyclosporine A shows most of the activation at low and high doses and early and middle time points, while thioacetamide shows most of the activation at low doses at all the time points (Fig. 3).

### Effect of time and dose on the dynamic MOA

**Figure 4:**
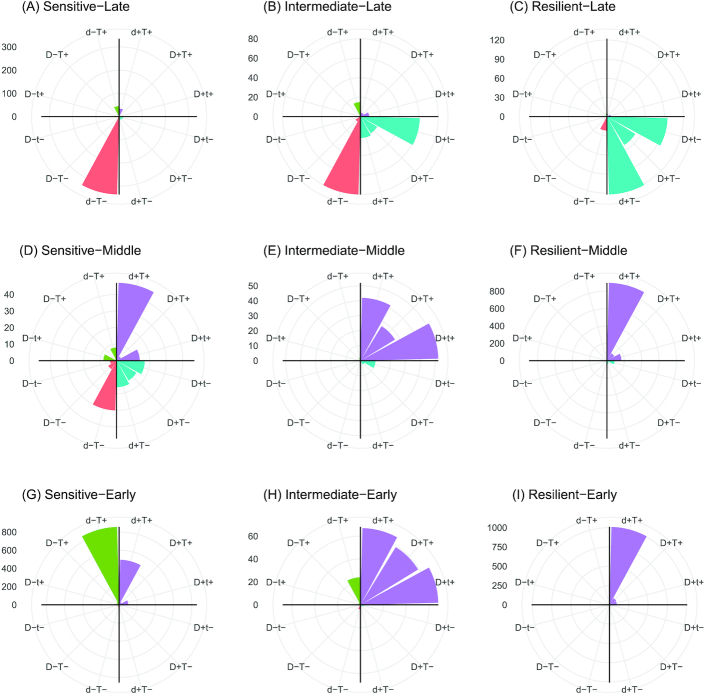
Cyclosporine A distribution of the number of responsive genes with respect to time and dose. The letters “d” and “t” stand for dose and time, respectively. In the label, a capital letter means that there is a stronger effect of a variable with respect to the other. If the 2 letters are both capitals, it means that the effect is the same. Concordance in the increase of the fold-change and dose/time is indicated with a plus sign, and decrease, with a minus sign. For cyclosporine A, the effect of the dose is particularly strong for the intermediate-early and intermediate-middle genes (capital D in Figs 4H and E), while the effect of time is more evenly distributed across the possible combinations. The early and middle genes mostly show an increase of the log fold-change with respect to the time (E, F, H, and I), whereas the fold-change of late genes decreases over time. Considering the dose, the log fold-change of sensitive-late genes mainly decreases as the dose increases (D and A). Differently, the intermediate and resilient genes show an increasing trend with respect to the dose (C, E, F, H, and I).

**Figure 5:**
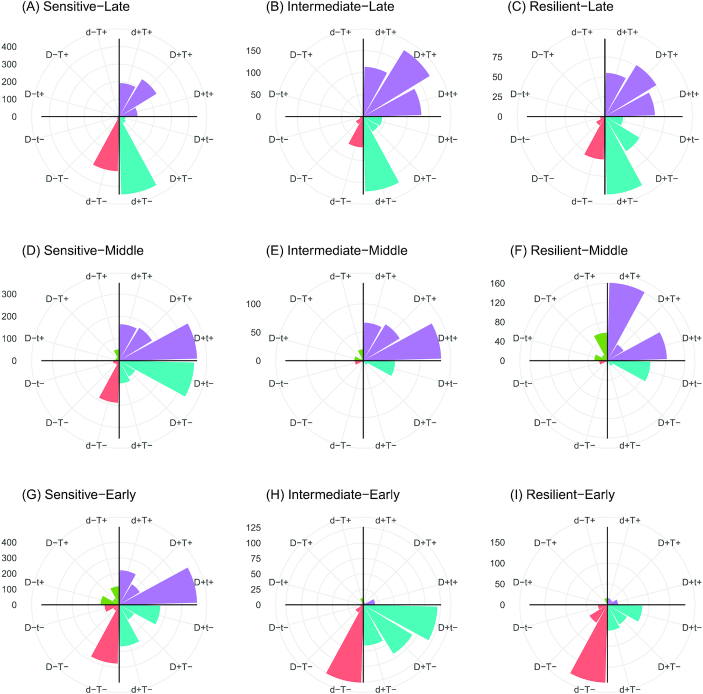
Thioacetamide distribution of the number of responsive genes with respect to time and dose. The letters “d” and “t” indicate dose and time, respectively. In the label, a capital letter means that there is a stronger effect of a variable with respect to the other. If the 2 letters are both capitals, it means that the effect is comparable. Concordance in the increase of the fold-change and dose/time is indicated with a plus sign, and decrease, with a minus sign. For thioacetamide, the effect of the time on the dynamic dose-responsive genes is stronger than the effect of the dose (capital T in A–C, F, H, and I). The increase of time corresponds to an increase in the gene expression in the intermediate-middle and resilient-middle genes (E and F). An opposite effect is visible on the intermediate-early and resilient-early genes (H and I). In all the other sets of genes the positive and negative effect of the time on the fold-change is balanced (A–D and G). With respect to the dose, intermediate-middle and resilient middle genes mainly show a direct correlation between the increase of the fold-change and the dose (E and F); the same effect can also be observed in the early, sensitive-middle, and late genes (violet and green bars in A–D, G, and green bars in H and I) even though some of them are negatively affected by the doses (red bars in A–D and G–I).

Even though the labels that TinderMIX assigns inform on the gene POD, they do not give insights on the relative impact of the dose and time on the variation of the log fold-change. To dissect these effects and the relative contribution of dose and time to the gene alteration, TinderMIX weighs their effect in the time-dose effect maps (Fig. 1B). Indeed, by studying the direction of the gradient in each pixel of the DDRA (Fig. 1C), we are able to assess the contribution of time and dose to the DDR behaviour of the gene expression and whether its effect is positive or negative (Fig. 1E). Moreover, TinderMIX generates a radar plot that summarizes the relative effect of dose and time onto the DDRG as well as their direction (Figs 4 and 5). There are different scenarios where it is useful to recognize whether the genes are more prominently affected by the dose or the time. For example, genes for which the exposure has a predominant effect might be more sensitive to the dose. On the other hand, some genes might be under complex regulatory mechanisms, some of which could be secondary to the exposure itself. Thus, their expression is not only altered in a dose-dependent manner but also kinetically modulated along the time. Indeed, in both cyclosporine A and thioacetamide, late genes undergo a stronger effect of time than of the dose (Figs 4 A–C and 5 A–C), as expected because transcriptomic alterations happening 24 h after the exposure are more likely to be due to regulatory processes happening downstream from the primary drug-induced response. On the other hand, early and middle genes appear to be more affected by the dose, especially in the case of thioacetamide, a compound with a more pronounced known toxic potential (Fig. 5 D, E, G, and H). Therefore, our tool provides better insights about the exposure MOA on a biological system, by providing both quantitative and qualitative estimation of the perturbation with respect to time and dose.

### Pathway enrichment analysis

**Figure 6:**
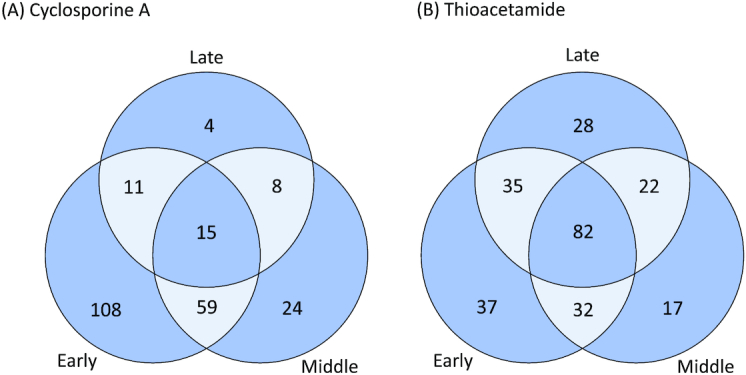
Venn diagram of the number of enriched pathways for each time category (early, middle, late) for the dynamic dose-responsive genes of cyclosporine A (A) and thioacetamide (B).

To characterize the biological processes underlying the POD labels assigned to the DDRG, we performed KEGG enrichment analysis of the genes belonging to the 9 label categories previously identified (Additional Files 5 and 6). We further grouped the enriched pathways on the basis of the time of activation to draw a kinetic map of the events in the exposure time (Fig. 6). Cyclosporine A is a known immunosuppressant drug, with a low toxic potential. Indeed, at early time points, 108 deregulated pathways were found (Fig. 6A), while, at middle and late time points, only a few deregulated pathways (24 and 4, respectively) were obtained. Cyclosporine A is known to inhibit the activation of T lymphocytes by blocking the activity of calcineurin phosphatase [30]. In fact, several interleukins and chemokine-driven immune system mechanisms were found significantly deregulated at early time points. In particular, Th1 and Th2 cell differentiation pathways were found altered. Among the main effectors of such pathways *Cd4*, one of the main markers of T lymphocytes, was labelled by TinderMIX as a sensitive-early DDR gene. On the other hand, thioacetamide exposure has been associated with liver toxic effects and inflammatory cell infiltration [31]. As might be expected for a more toxic drug, more deregulated pathways at any time point were retrieved in our analysis (Fig. 6B). Among the ones enriched at early time points, infectious disease pathways such as hepatitis B and C, as well as the apoptosis pathway, were enriched and both apoptotic and necrosis-related genes were up-/downregulated, such as *Fadd, Fas, Bad*, and *Bid*. Consistently with a hepatotoxic induced effect, the *Aldh2* gene, which is known to be altered in patients with chronic hepatitis and non-alcoholic cirrhosis, was found also deregulated in intermediate pathways [32].

### Visual inspection of the gene maps

**Figure 7:**
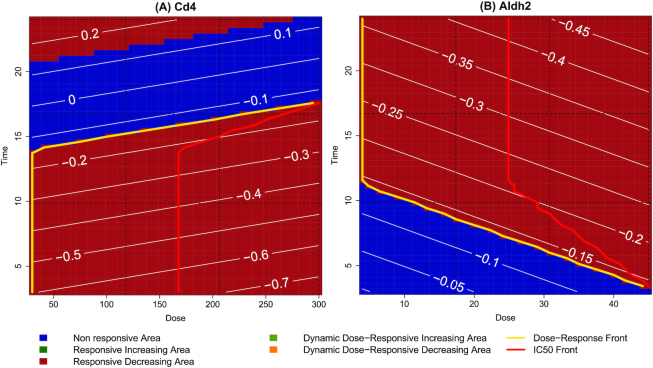
Time-dose effect maps of the *Cd4* (A) and *Aldh2* (B) genes.

To complement the information given by the POD labels, the time-dose effect maps of the DDRG allow visualization of the whole kinetics of the log fold-changes in the joint dose-time space. Furthermore the levels of the contour plots specify whether the gene is up- or downregulated. The effect maps of the previously identified genes are described as an example. As we can see from Fig. 7A, *Cd4* is marked as sensitive-early, and it is downregulated at early time points (log fold-change in [−0.7, −0.4]). Furthermore, the log fold-change increases at middle time points (log fold-change in [−0.4, −0.1]) and is eventually upregulated at late time points (log fold-change in [0.1, 0.2]). The expression pattern of this gene changes over time in concordance with the known MOA of cyclosporine A [30]. On the other hand, the *Aldh2* gene is labelled as sensitive-middle because the DDRA begins at the lowest doses and middle time points (Fig. 7B). It does not show any activity at early time points with low and middle doses, while it is dynamically dose-dependent and downregulated already at early time points with high doses. Its strength of downregulation increases with both time and dose (log fold-change in [−0.15, −0.45]). The overall downregulation visible in the map has already been reported in previous studies demonstrating that, in rats, thioacetamide can directly inhibit the Aldh2 isoenzyme [33].

## Conclusion

In this study, we proposed TinderMIX, a novel methodology for the joint time and dose modelling of toxicogenomics data. TinderMIX consists of different steps that combine polynomial model fitting, active region extraction, and pathway enrichment analysis to identify genes with joined dose and time patterns of responsiveness. TinderMIX allows the user to study the dynamic behaviour of the genes in the joint dose-time space, by interpolating the omics feature levels and filling the gaps between the doses and time points tested in the experiment. TinderMIX automatically assigns to the responsive genes an activation label that specifies the joint dose and time POD of the gene and estimates the strength of the effect of time and dose on each gene activation. Moreover, it allows graphical inspection of the gene maps as contour plots. Each gene map can give insights into the dynamic and dose-dependent shape of the gene log fold-change. It easily shows the POD of the gene, and it highlights the monotonic trend of the responsive area. The TinderMIX methodology also helps in grouping the genes on the basis of the activation label and identifying the set of pathways associated with each group in order to better characterize the underlying biological mechanisms. In conclusion, TinderMIX can be used to investigate the point of departure of genes with respect to dose and time point upon chemical exposure with an integrated analytical approach.

## Availability of Source Code and Requirements

The TinderMIX method is available in the form of an R package, which is available via a git repository:

- Project name: TinderMIX
- Project home page: https://github.com/grecolab/TinderMIX
- Operating system(s): Platform independent
- Programming language: R
- Other requirements: Java
- License: GNU GPL (version 3 or greater)
- RRID:SCR_018364

## Availability of Supporting Data and Materials

The data used to showcase the TinderMIX methodology are available in the git repository at https://github.com/grecolab/TinderMIX/tree/master/sample_data. Further supporting data and snapshots of our code are openly available in the GigaScience repository, GigaDB [34].

## Additional Files

**Additional File 1:** TinderMIX pseudo-code

**Additional File 2:** List of dynamic dose-responsive genes for cyclosporine A. The file contains the following information: (1) dose_time_comparison: specifies whether the activation is more dependent on the dose or the time; (2) Gene Description: is a text description of the gene; (3) Gene Symbol; (4) Joint Label: is the POD label; (5) gene_sign: specifies whether the gene activation is increasing or decreasing with respect to the dose; (6) MeanFC: mean log fold-change of the gene in the POD area; (7) adj.pval: adjusted *P*-value of the fitted polynomial model

**Additional File 3:** List of dynamic dose-responsive genes for thioacetamide. The file contains the following information: (1) dose_time_comparison: specifies whether the activation is more dependent on the dose or the time; (2) Gene Description: is a text description of the gene; (3) Gene Symbol; (4) Joint Label: is the POD label; (5) gene_sign: specifies whether the gene activation is increasing or decreasing with respect to the dose; (6) MeanFC: mean log fold-change of the gene in the POD area; (7) adj.pval: adjusted *P*-value of the fitted polynomial model

**Additional File 4:** Sensitivity analysis of the activation threshold

**Additional File 5:** List of pathways for cyclosporine A

**Additional File 6:** List of pathways for thioacetamide

## Abbreviations

ANOVA: analysis of variance; BMD: benchmark dose analysis; DDR: dynamic dose response; DDRA: dynamic dose responsive area; DDRG: dynamic dose responsive gene; IC50: half maximal inhibitory concentration; KEGG: Kyoto Encyclopedia of Genes and Genomes; MOA: mechanism of action; Open TG-GATEs: Open Toxicogenomics Project-Genomics Assisted Toxicity Evaluation System; POD: point of departure; TinderMIX: Time-Dose Integrated Modelling of Omics Data.

## Competing Interests

The authors declare that they have no competing interests.

## Funding

This study was supported by the Academy of Finland (grant number 322761) and the EU H2020 NanoSolveIT project (grant number 814572).

## Authors' Contributions

Conceptualization: A.S., D.G., L.A.S., Data curation: A.S., Formal Analysis: A.S., Funding acquisition: D.G., Methodology: A.S., M.F., M.P., Project administration: D.G., A.S., Software: A.S., M.F., M.P., Supervision: D.G., Visualization: A.S., Writing – original draft: A.S., M.F., G.d.G., A.F., D.G. Writing - review and editing: A.S., M.F., G.d.G., A.F., L.A.S., M.P., D.G. All authors have read and agreed to the published version of the manuscript.

## Supplementary Material

giaa055_GIGA-D-20-00057_Original_Submission

giaa055_GIGA-D-20-00057_Revision_1

giaa055_GIGA-D-20-00057_Revision_2

giaa055_Response_to_Reviewer_Comments_Original_Submission

giaa055_Response_to_Reviewer_Comments_Revision_1

giaa055_Reviewer_1_Report_Original_Submission Diana Hendrickx -- 3/5/2020 Reviewed

giaa055_Reviewer_1_Report_Revision_1 Diana Hendrickx -- 4/25/2020 Reviewed

giaa055_Reviewer_2_Report_Original_Submission Haralambos Sarimveis -- 3/30/2020 Reviewed

giaa055_Supplemental_Files
