# Supplementary material for: TinderMIX: Time-dose integrated modelling of toxicogenomics data
Source: Gigascience. 2020 May 25;9(5):giaa055. doi: 10.1093/gigascience/giaa055 (PMC7247400; doi:10.1093/gigascience/giaa055)
Supplement: giaa055_GIGA-D-20-00057_Original_Submission [file giaa055_giga-d-20-00057_original_submission.pdf]

GigaScience

TinderMIX: Time-Dose INtegrated moDelling of toxicogenomics data

--Manuscript Draft--

**Manuscript Number:**	GIGA-D-20-00057
**Full Title:**	TinderMIX: Time-Dose INtegrated moDelling of toxicogenomics data
**Article Type:**	Research
**Funding Information:**	Horizon 2020 Framework Programme (814572) — Prof Dario Greco<br>Terveyden Tutkimuksen Toimikunta (322761) — Prof Dario Greco
**Abstract:**	Background: Omics technologies have been widely applied in toxicology studies in order to investigate the effects of different substances on exposed biological systems. A classical toxicogenomic study consists in testing the effects of a compound at different dose levels and different time points. The main challenge consists in identifying the gene alteration patterns that are correlated to doses and time points. Existing methods for toxicogenomics data analysis allow studying molecular alteration after the exposure (or treatment) at each time point individually. However, this kind of analysis is not able to identify dynamic (time-depended) events of dose responsiveness.<br>Results: We propose TinderMIX, an approach that simultaneously models the effects of time and dose on the transcriptome to investigate the course of molecular alterations exerted in response to the exposure. Starting from gene log-fold-change, TinderMIX fits different integrated time and dose models to each gene, selects the optimal one, and computes its time and dose effect map; then a user-selected threshold is applied to identify the responsive area on each map and verify if the gene shows a dynamic (time-dependent) and dose-dependent response; eventually, responsive genes are labelled according to the integrated time and dose point of departure (POD).<br><br>Conclusions: To showcase the TinderMIX method, we analyzed two drugs from the Open TG-GATEs dataset, namely cyclosporin A and thioacetamide. We first identified the dynamic dose-dependent mechanism of action (MOA) of each drug and compared them. Our analysis highlights that different time and dose integrated POD recapitulates the toxicity potential of the compounds as well as their dynamic dose-dependent MOA.
**Corresponding Author:**	Dario Greco<br>Tampereen Yliopisto<br>Tampere, FINLAND
**Corresponding Author Secondary Information:**	
**Corresponding Author's Institution:**	Tampereen Yliopisto
**Corresponding Author's Secondary Institution:**	
**First Author:**	Angela Serra
**First Author Secondary Information:**	
**Order of Authors:**	Angela Serra<br>Michele Fratello<br>Giusy del Giudice<br>Laura Aliisa Saarimäki<br>Michelangelo Paci<br>Antonio Federico<br>Dario Greco
**Order of Authors Secondary Information:**	

Powered by Editorial Manager® and ProduXion Manager® from Aries Systems Corporation

Additional Information:	
**Question**	**Response**
Are you submitting this manuscript to a special series or article collection?	No
**Experimental design and statistics**<br><br>Full details of the experimental design and statistical methods used should be given in the Methods section, as detailed in our Minimum Standards Reporting Checklist. Information essential to interpreting the data presented should be made available in the figure legends.<br><br>Have you included all the information requested in your manuscript?	Yes
**Resources**<br><br>A description of all resources used, including antibodies, cell lines, animals and software tools, with enough information to allow them to be uniquely identified, should be included in the Methods section. Authors are strongly encouraged to cite Research Resource Identifiers (RRIDs) for antibodies, model organisms and tools, where possible.<br><br>Have you included the information requested as detailed in our Minimum Standards Reporting Checklist?	Yes
**Availability of data and materials**<br><br>All datasets and code on which the conclusions of the paper rely must be either included in your submission or deposited in publicly available repositories (where available and ethically appropriate), referencing such data using a unique identifier in the references and in the "Availability of Data and Materials" section of your manuscript.	Yes

Powered by Editorial Manager® and ProduXion Manager® from Aries Systems Corporation

Have you have met the above requirement as detailed in our Minimum Standards Reporting Checklist?	

Powered by Editorial Manager® and ProduXion Manager® from Aries Systems Corporation

Manuscript Click here to download Manuscript main_tindermix.pdf

GigaScience, 2020, 1–9

doi: xx.xxxx/xxxx
Manuscript in Preparation
Paper

PAPER

TinderMIX: Time-Dose INtegrated moDelling of toxicogenomics data

Angela Serra[1,2], Michele Fratello[1,2], Giusy del Giudice[1,2], Laura Aliisa Saarimäki[1,2], Michelangelo Paci[2], Antonio Federico[1,2] and Dario Greco[1,2,3,*]

[1]Faculty of Medicine and Health Technology, Tampere University, Tampere, Finland and [2]BioMediTech Institute, Tampere University, Tampere, Finland and [3]Institute of Biotechnology, University of Helsinki, Helsinki, Finland

[*]To whom correspondence should be addressed: dario.greco@tuni.fi

Abstract

Background: Omics technologies have been widely applied in toxicology studies in order to investigate the effects of different substances on exposed biological systems. A classical toxicogenomic study consists in testing the effects of a compound at different dose levels and different time points. The main challenge consists in identifying the gene alteration patterns that are correlated to doses and time points. Existing methods for toxicogenomics data analysis allow studying molecular alteration after the exposure (or treatment) at each time point individually. However, this kind of analysis is not able to identify dynamic (time-depended) events of dose responsiveness.
Results: We propose TinderMIX, an approach that simultaneously models the effects of time and dose on the transcriptome to investigate the course of molecular alterations exerted in response to the exposure. Starting from gene log-fold-change, TinderMIX fits different integrated time and dose models to each gene, selects the optimal one, and computes its time and dose effect map; then a user-selected threshold is applied to identify the responsive area on each map and verify if the gene shows a dynamic (time-dependent) and dose-dependent response; eventually, responsive genes are labelled according to the integrated time and dose point of departure (POD).
Conclusions: To showcase the TinderMIX method, we analyzed two drugs from the Open TG-GATEs dataset, namely cyclosporin A and thioacetamide. We first identified the dynamic dose-dependent mechanism of action (MOA) of each drug and compared them. Our analysis highlights that different time and dose integrated POD recapitulates the toxicity potential of the compounds as well as their dynamic dose-dependent MOA.

Key words: toxicogenomics, gene expression, integrated modeling, dose-response, time course, BMD, POD, point of departure, dynamic dose-dependent, mechanism of action, MOA.

Introduction

Toxicogenomic methods are widely used for the assessment of chemical hazards and environmental health [1]. Omics technologies have been broadly accepted and recognized as efficient and reproducible tools to study the effects of chemical exposures on different organisms [2]. In particular, transcriptomics technologies allow the investigation of gene expression patterns of thousands of genes after exposure to a substance [3] and they have been widely used in toxicogenomics [4, 5, 6, 7].

Direct effects of chemical insults are generally expected to follow a monotonic dose-response alteration resulting in a more impactful effect as the dose increases until a plateau is reached[8]. In classical toxicology, this is observed as an in-

Compiled on: February 27, 2020.
Draft manuscript prepared by the author.

creasing number of deceased animals or any other measurable apical endpoint. A similar effect can be appreciated in gene expression alteration. Since the changes in gene expression occur much earlier than apical endpoints, transcriptomic data can be used as a complementary approach to estimate relevant points of departure (POD) and hence provide valuable information for the toxicological assessment [9, 10].

Until now, the no-observed-adverse-effect-level (NOAEL)[11] and the benchmark dose (BMD)[12] methods have been used to help the interpretation of transcriptomic dose-response data and to derive transcriptomic PODs. In particular, by modelling the patterns of dose-responsiveness of gene expression, the BMD method provides an estimate of the lowest dose of a chemical able to induce a significant change in biological activity.

However, assaying the mechanism of action of exposure at multiple time points is valuable to highlight useful information on the kinetic molecular responses of a biological system. For example, the experimental setup for the in vivo studies in the Open TG-GATEs involves animal treatment in which each drug is tested at three doses (low, medium and high) at multiple time-points [7]. Thus, one major challenge is the interpretation of these toxicogenomics data, especially for identifying patterns of alterations of the gene expression that are correlated both to the dose levels and the time points. Different methods have been proposed to identify genes that show a dose-response effect [13, 14] or to study the gene expression dynamics at individual time points [15, 16]. However, this kind of approach does not allow an easy interpretation of the time-dependent dynamics happening after the exposure and the molecular adaptation process. To date, few efforts have been done to propose a methodology able to identify sets of genes that show similar expression patterns with respect to both dose and time [17].

In this work, we propose a new computational framework, TinderMIX, for dose- and time-dependent gene expression analysis that aims to combine dimensionality reduction, BMD analysis, and polynomial fitting to find groups of genes that show a dynamic dose-response (DDR) behaviour. The integrated modelling used in TinderMIX allows us to interpolate the continuous joint dose-time space and predict the molecular alteration values for the doses and time-points not included in the original experiment. Moreover, our approach is importantly able to inform on POD in both the dimensions of the doses and time points, hence resolving at once two analytical tasks that, thus far, have only been carried out subsequently to each other.

To illustrate our methodology we analyzed the gene expression data for cyclosporine A and thioacetamide from the Open TG-GATEs database.

Materials and Methods

The TinderMIX methodology proposed in this study starts from the sample-wise log-fold-change of the genes and is able to identify which of them show a DDR behaviour and to estimate

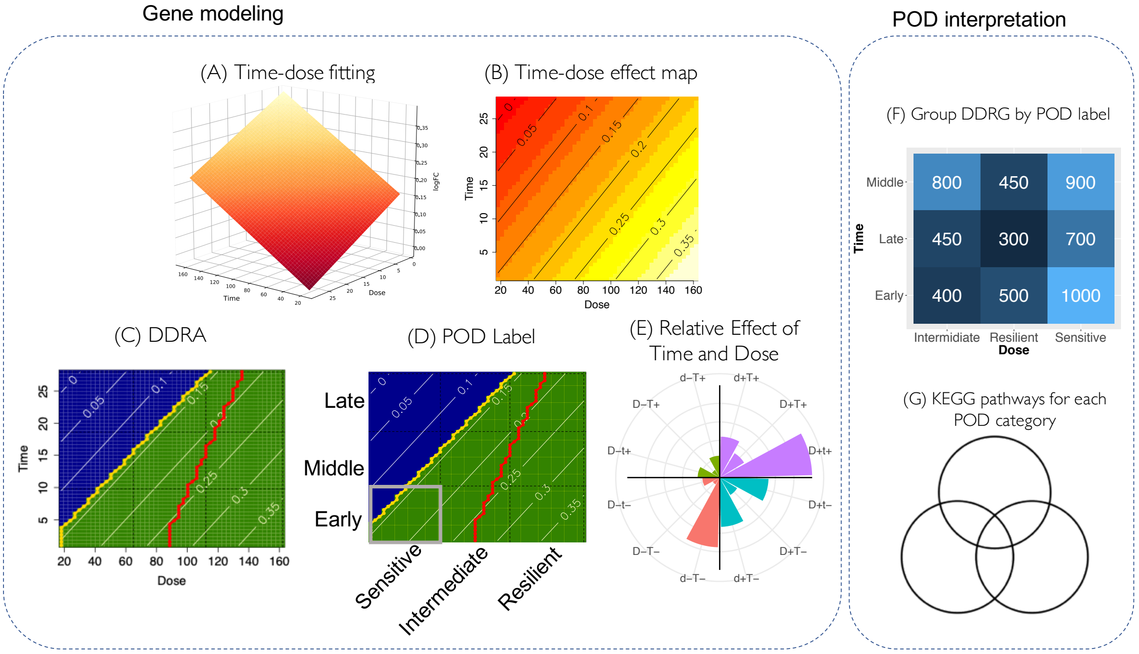

Figure 1. TinderMIX methodology. The TinderMIX methodology is composed of several steps that are grouped into gene modeling and POD interpretation. First, starting from the pairwise log-fold-change of each gene a 3D polynomial model is fitted (A). Second, the 3D model is mapped in its 2D effect map by means of contour plots. The lines in the contour plots describe the shape of the 3D model by showing the portion of the space where the fitted model has a constant log-fold-change, indicated by the value on the lines. A positive value means that the gene is up-regulated otherwise it is dow-regulated (B). Third, the dynamic-dose-responsive area (DDRA) is identified. Starting from a user-defined activity threshold, the time-dose effect map is divided into regions of different colors. The blue region is the part of the map where the log-fold-change is lower than the threshold, thus marked as a non-active area. The green and red areas are the active ones. In particular, the green area is characterized by an increase of the log-fold-change when the dose increases, while the red area presents a decrease of log-the fold-change when the dose increases. A gene is considered DDR, if there is a DDRA in the activity map, with a monotonic behavior of the log-fold-change from a dose to the highest one tested. In the map, the front of the dose-responsive area is marked with a yellow line. Moreover, a red line is used to mark the IC50 front, which is the line connecting the set of doses (one for each time point) that specifies the 50% of gene activity (C). Fourth, based on the first dose and time of activation the gene is labelled with an activation label that specifies its dose and time POD. The time-dose effect map is divided into a 3 by 3 grid. The sections of the dose axis were named “sensitive”, “intermediate”, and “resilient”, while for the time axis the labels “early”, “middle”, and “late”, were assigned. The final label was then obtained by identifying the earliest and most sensitive point of activation and concatenating the dose and time of the single labels (D). Fifth, the effect of the dose and time in the DDRA is compared (E). The second part of the analysis consists of the POD interpretation. Thus, the DDRGs identified are grouped by their POD labels (F) and eventually a pathway enrichment analysis for the genes in each category is performed (G).

their joint dose-time POD. The methodology is composed of multiple steps that can be grouped into two parts: the genes modelling with POD identification (Figure 1) that is executed for every gene in the dataset, and the POD interpretation of the dynamic dose-responsive genes (DDRG) identified in the first part (Figure 1).

As for the classical BMD analysis, the first step of the gene modeling analysis (Figure 1A) consists of fitting different polynomial functions to each gene log-fold-change and identifying the optimal one by using a Nested Model Hypothesis test [18]. The difference with the classical BMD analysis performed so far is that the fitted models are three-dimensional (3D) functions in the space of the dose, log-fold-change, and time. Indeed, so far, the BMD analysis has mainly been performed in the dose and log-fold-change space for each time point individually. Afterward, the optimal 3D model is mapped in the dose-time dimensional space by means of contour plots (Figure 1B). A monotonically increasing or decreasing (with respect to the doses) dynamic dose-responsive area (DDRA) is identified starting from a user-specified activation threshold. (Figure 1C). If this DDRA is present, the gene is considered DDR and his POD is identified based on the first dose level and activation time (Figure 1D). Moreover, the effect of dose and time in the DDRA is compared in order to understand if the modifications in the log-fold-changes values are due to a positive or negative effect of the time and dose and also to understand which of the two variables contribute most(Figure 1E). Indeed, if the time has a stronger effect compared to the dose we can hypothesize that the effect under analysis can be an adaptation process, on the other hand, if the dose contributes the most we can hypothesize that we are observing an effect directly correlated to the exposure. In the second part of the analysis, the DDR genes identified can be grouped according to their POD labels (Figure 1F). Eventually, overrepresented pathways can be calculated for each gene category (Figure 1G).

Time-dose Fitting

For every gene, linear regression models including 1st, 2nd and 3rd order polynomials (equations 1-3) of the explanatory variables (log-fold-change) are fitted.

$$LFC = \beta_0 + \beta_1 Dose + \beta_2 Time \tag{1}$$

$$\begin{aligned} LFC = {} & \beta_0 + \beta_1 Dose^2 + \beta_2 Time^2 \\ & + \beta_3 (Dose \times Time) + \beta_4 Dose + \beta_5 Time \end{aligned} \tag{2}$$

$$\begin{aligned} LFC = {} & \beta_0 + \beta_1 Dose^3 + \beta_2 (Dose^2 \times Time) \\ & + \beta_3 (Dose \times Time^2) + \beta_4 Time^3 + \beta_5 Dose^2 \\ & + \beta_6 Time^2 + \beta_7 (Dose \times Time) + \beta_8 Dose + \beta_9 Time \end{aligned} \tag{3}$$

The fitting was performed by using the R lm function from the stats package [19]. The best-fitting model is selected performing a Nested Model Hypothesis test [18]. The test is performed using the R function ANOVA, which takes a list of models as an input. Each model in the list is considered as the "full" model with respect to the previous "restricted" model in the list, where a number of partial regression coefficients are set to zero. In other words, a model is restricted, with respect to the full model, in the sense that it does not consider one or more explanatory variables. For each pair of subsequent models in the list, we test the significance of the amount of variance explained by a subset of predictors of the restricted model, compared to the variance explained by all the variables of the full model. We also add to the test, at the head of the list of models, an implicit constant model in which all the partial regression coefficients are zero, except for the intercept β_0. This is done to assess the quality of the fit in the first place. If the p-values corresponding to testing the models in Eq. 1-3 against the constant model are not significant, that gene is not considered as having a dose-response effect. If, on the other hand, there is a significant amount of variance explained by any of the models in Eq. 1-3, then we return the fitted model corresponding to the lowest p-value.

Time-dose effect map prediction and Dynamic dose-responsive genes identification

For each gene, the selected model is used to predict an activity map with the values of a smooth log-fold-change function on a grid of 50 x 50 points covering the entire range of doses and time-points tested. This map is represented as a contour plot and used to identify the dose-response area. A desired activity threshold corresponding to a 10% increase/decrease with respect to controls is set to identify the responsive area of each gene time-dose effect map. If a gene does not show an activity satisfying the threshold it is removed from the analysis. A gene is also removed from the analysis if the responsive area does not include the highest dose of the experimental setting. For the genes passing the previous filtering, the gradient of the smooth log-fold-change function is computed at each point of the 50 x 50 grid. Each point of the grid is segmented according to if its connected components show a monotonically (with respect to the whole dose range) increment or decrement of the log-fold-change values. If only one component, either with increasing or decreasing behaviour, is present in the map and it contains the highest dose, then the component is selected as the candidate DDRA. However, if the highest dose is not contained in the area, then the gene is considered not responding and removed from the analysis. On the other hand, if multiple components with different increasing or decreasing behaviour, are present in the map, one of the two is selected as candidate dose-responsive area, according to the criteria described in additional file 1. In particular, each candidate region is weighted according to the following criteria:

$$r_i = n_p + n_{tp-md} - m_d, i \in \{r_1, \ldots, r_n\} \tag{4}$$

Where n_p is the number of points included in the region, n_{tp-md} is the number of time-points (rows in the gene map) that are included in the candidate region that include the highest dose and m_d is the minimum dose covered by the candidate region and n is the number of candidate regions found in the gene maps. The optimal region is selected as the one maximizing the score. The active region is further reduced, by removing time-points that are still active (log-fold-change > 1.1) outside the optimal region but with a non-monotonic behaviour compared to the one inside the optimal region.

We identify the external borders of the segmented responsive area using a trace-boundary algorithm. The dose-responsive front is identified as the smallest dose present in the responsive area for each timepoint. Moreover, the IC50 front is also computed as the doses that give 50% of changes in the log-fold-change at every time-point.

The dose-time space is partitioned into 3×3 regions. Where each dose can be labelled as sensitive, intermediate or resilient and each time is labelled early, middle or late. The final label was then obtained by identifying the earliest and most sensitive point of activation and concatenating the dose and time of the

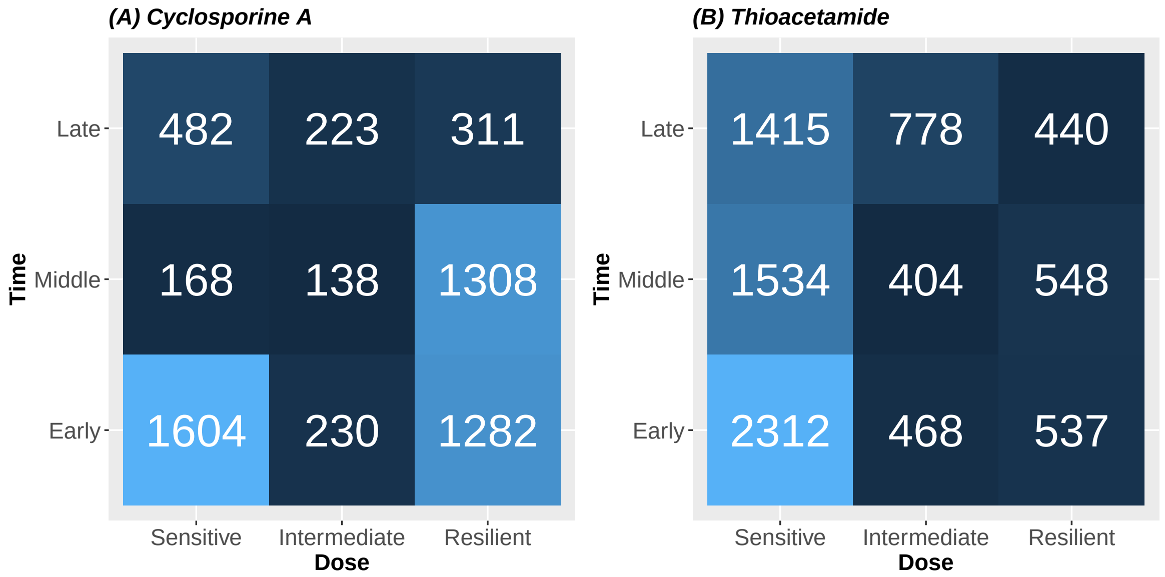

Figure 2. The number of dose-responsive genes for each gene category in Cyclosporine A (A) and Thioacetamide (B).

single labels. For example, a gene with label sensitive-early is a gene that shows a response already at low doses and early time points. On the other hand, a resilient-late gene shows a response only at high doses and late time points.

Time and dose influence by means of gradient

Once the dynamic dose-responsive area is identified, the influence of the time on the fold-change is studied by analyzing the vector field of gradients in the region. For each point, the magnitude and the angle of the gradient are computed and used to evaluate the time-dose response score as follows:

$$td_s = \frac{1}{\sum_{i=1}^{n} mod_i} \times (\sum_{i=1}^{n} g_i \times mod_i) \quad (5)$$

Where g_i and mod_i are the angle and magnitude of the gradient in the $i-th$ point in the dynamic dose-responsive region and n is the number of points in the same region. This score can be used to categorize the genes based on the dose and time effect on their fold change. According to the time-dose response score, the active genes are divided into four groups, corresponding to the four quadrants of the cartesian space. In the first quadrant the fold change increases with both time and dose ($0 < td_s \leq 90$); in the second quadrant, the fold change increases with time, but decreases with dose ($90 < td_s \leq 180$); in the third quadrant, the fold change decreases with both time and dose ($180 < td_s \leq 270$); in the fourth quadrant the fold change increases with dose and decreases with time ($270 < td_s \leq 360$). The four quadrants can be further dissected in three smaller sectors: one in which the dose has a stronger effect than the time, one in which they have the same effect and one in which the time has a stronger effect than the dose. We assign a label to each gene composed by the letters d and t standing for dose and time respectively and a positive or negative sign. If the influence of one of the two components is stronger than the other, this is highlighted by using capital letters. For example, the label $d+T+$ stands for fold change increasing both with dose and time with a stronger effect from the time.

Enrichment of biological pathways

KEGG enrichment analysis is performed by using the methodology implemented in the FunMappOne tool ??. Pathways were considered significantly enriched if they have a corrected p-value lower than 0.05.

Dataset collection

Pathological events registered in rats after drug exposure were downloaded from the Open TG-GATEs database (https://dbarchive.biosciencedbc.jp/en/open-tggates/download.html). For every drug pathological score was computed by counting the number of events occurring after the exposure normalized by the number of events in the controls. Cyclosporine A and thioacetamide were selected as representative candidates for drugs with low and high toxic impact, respectively.

Raw data microarray transcriptomic data for Cyclosporine A and thioacetamide exposure were downloaded from the Open TG-GATEs dataset. Each dataset consists of 48 samples, of which 12 are unexposed controls and 12 are replicates for each dose level. Cyclosporine A was tested with doses 30, 100, and 300 mg/kg, while Thioacetamide with doses 4,15 and 45 mg/kg. In both cases, samples were harvested at four different time points (3, 6, 9 and 24 hours). The raw data were imported into R v. 3.4 by using the justRMA function from the Bioconductor utilities [20] to annotate probes to Ensembl genes (by using the rat2302rnensgcdf (v. 22.0.0) annotation file from the brainarray website (http://brainarray.mbni.med.umich.edu/), and quantile normalize the resulting expression matrix. Next, the experimental batch effects due to technical variables were estimated and removed using the ComBat algorithm implemented in the Sva package [20, 21]. For each pair of dose and time-point, all the pairwise log-fold-changes for each gene were computed as the difference between the log2 expression values of each pair of treated and control samples. In this way, we obtained 108 pair-samples and 11721 genes to be used in the TinderMIX analysis.

Results and Discussion

We developed a novel dose and time integrative modelling strategy for transcriptomics data able to identify molecular features with a dynamic dose-responsive alteration pattern (Figure 1). We showcase our methodology by analyzing in vivo gene expression data for two drugs (cyclosporine A and thioacetamide) available in the Open TG-GATEs dataset.

Dynamic dose-dependent mechanism of action (MOA)

The TinderMIX methodology allows to study the distribution of the gene log-fold-change with respect to both dose and time. For this purpose, TinderMIX implements a strategy similar to the classical BMD analysis[12] but translated into a three-dimensional space.

Table 1. The number of dynamic dose-responsive genes. The DDR is the number of dynamic dose-responsive genes. Linear is the number of DDR that is fitted by a liner, poly2 and poly3 are the numbers of DDR genes that are fitted by a linear, 2nd and 3rd order polynomial function respectively.

Drug	DDR	Linear	Poly2	Poly 3
cyclosporine A	5746	1362	4164	220
thioacetamide	8436	2352	1031	5053

For every gene, a linear, second, and third-order polynomial models are fitted (Figure 1A). The optimal model is selected as the one with adjusted goodness-of-fit p-value lower than 0.01 and best modelling performance according to the nested model hypothesis test, as performed by ANOVA. Furthermore, for the genes that pass the goodness-of-fit filter-

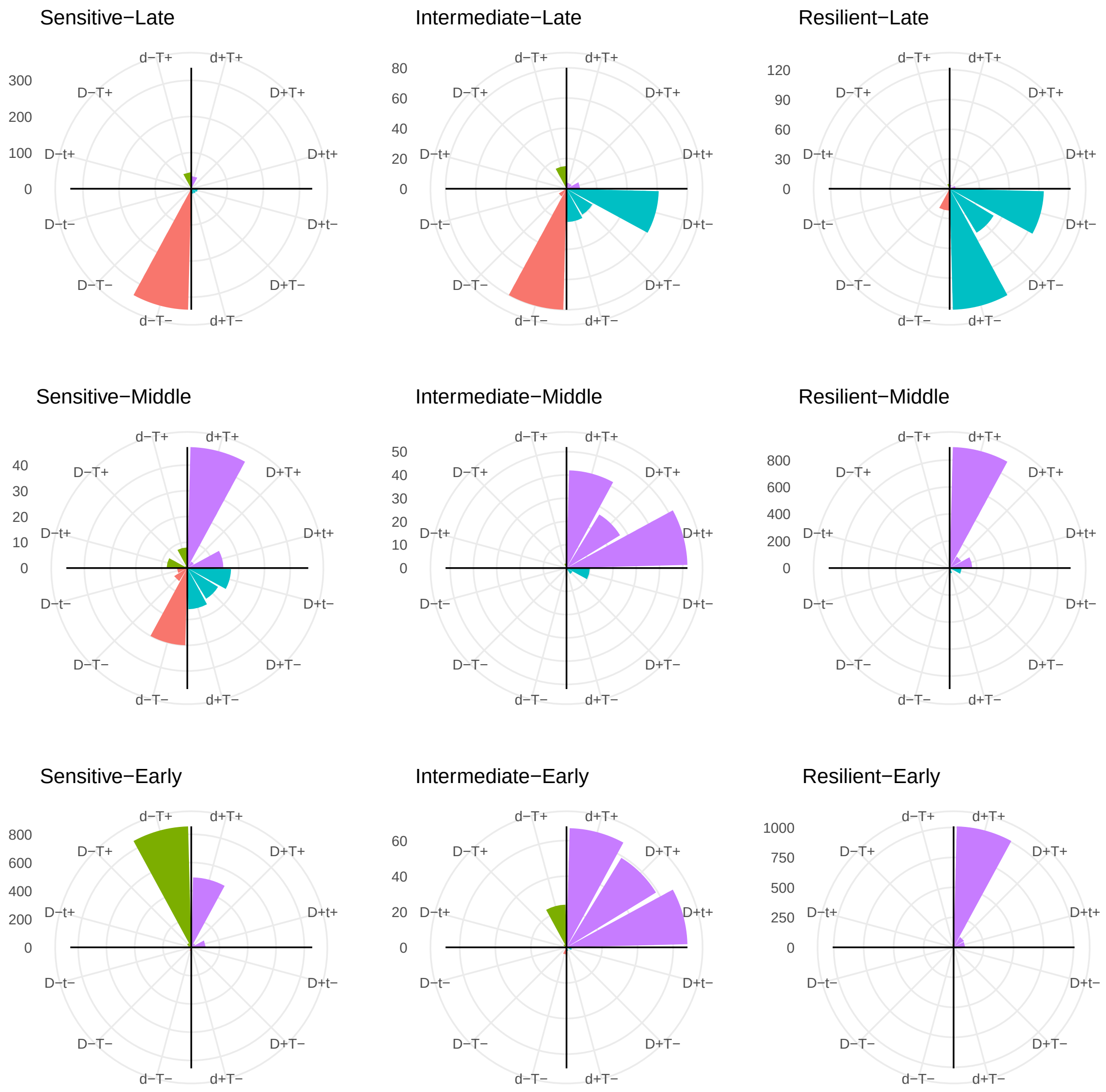

Figure 3. cyclosporine A distribution of the number of responsive genes with respect to time and dose. The letters d and t stand for dose and time respectively. In the label, a capital letter means that there is a stronger effect of a variable with respect to the other. If the two letters are both capitals it means that the effect is the same. Concordance in the increase of the fold change and dose/time is indicated with a +, while decrease with a -. For cyclosporine A, the effect of the dose is particularly strong for the intermediate-early and intermediate-middle genes (capital D in Figures 2H-2E), while the effect of time is more evenly distributed across the possible combinations (Figure 2). The early and middle genes mostly show an increase of the log-fold-change with respect to the time (Figures 2E, 2F, 2H, 2I), whereas the fold-change of late genes decreases over time. Considering the dose, the log-fold-change of sensitive-middle and sensitive-late genes mainly decreases as the dose increases. (Figures 2D, 2A) Differently, the intermediate and resilient genes show an increasing trend with respect to the dose (Figures 2C, 2E, 2F, 2H, 2I).

ing, their dynamic dose-responsiveness is investigated. Hence, TinderMIX maps the 3D optimal model in its corresponding time-dose effect map by contour plots (Figure 1B). This step allows identifying the DDRA, by iterating the concept of dose-responsiveness on each time point (Figure 1C). We consider a gene to be dose-dependently altered if its response is monotonic throughout all the doses at any time point. Given the complex kinetics of gene expression, interpolating the behaviour of the genes between the tested doses increases the robustness of dose-response modelling when multiple time points are assayed.

Starting from a standard gene activation threshold of 10%, TinderMIX identified 5,746 and 8,436 dose-responsive genes in cyclosporine A and thioacetamide, respectively (Table 1). In the case of cyclosporine A, most of the genes were fitted by the second-order polynomial model, while for thioacetamide by the third-order polynomial model (Table 1). This suggests that there is a predominant non-linear relationship between the log-fold changes, the dose levels, and the time-points.

The complete lists of dynamic dose-responsive genes for cyclosporine A and thioacetamide are available in additional files 2 and 3, respectively.

TinderMIX labelling for Point of departure

By identifying the first dose and time point present in the DDRA (Figure 1C), TinderMIX assigns to each DDRG an activity label (Figure 1D). The label provides information on the joint time-dose POD at a glance. For instance, the labels aid drawing a hypothetical sequence of events. The fact that a gene can re-

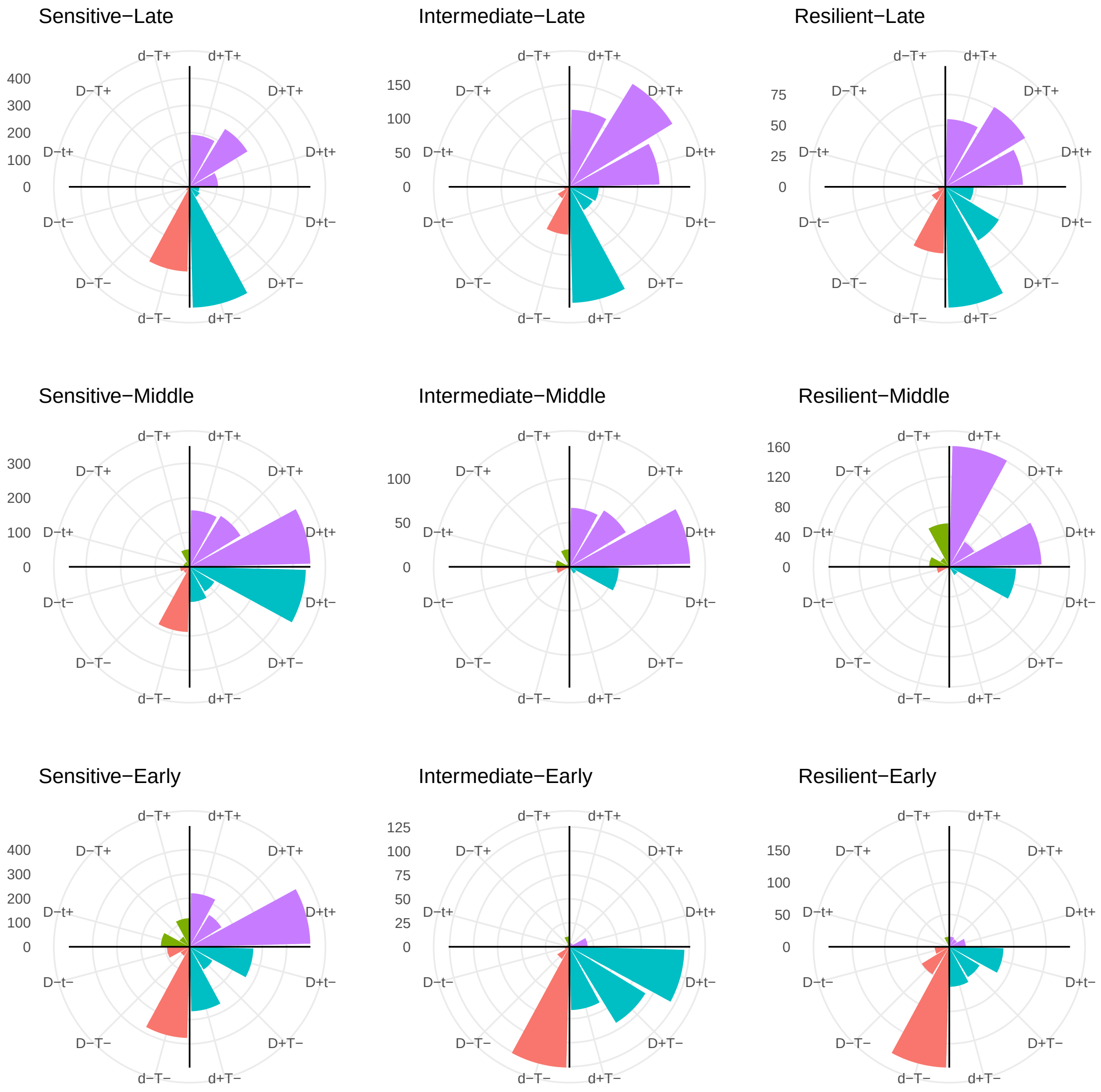

Figure 4. thioacetamide distribution of the number of responsive genes with respect to time and dose. The letters d and t indicate dose and time, respectively. In the label, a capital letter means that there is a stronger effect of a variable with respect to the other. If the two letters are both capitals it means that the effect is comparable. Concordance in the increase of the fold change and dose/time is indicated with a "+", while decrease with a "-". For thioacetamide, the effect of the time on the dynamic dose-responsive genes is stronger than the effect of the dose (capital T in Figures 3A, 3B, 3C, 3F, 3H, 3I). The increase of time corresponds to an increase in the gene expression in the intermediate-middle and resilient-middle genes (Figures 3E,3F). An opposite effect is visible on the intermediate-early and resilient early genes (Figures 3H and 3I). In all the other sets of genes the positive and negative effect of the time on the fold-change is balanced (Figures 3A, 3B, 3C, 3D, 3G). With respect to the dose, intermediate-middle and resilient middle genes mainly show a direct correlation between the increase of the fold-change and the dose (Figures 3E,3F), also the same effect can be observed in the early, sensitive-middle and late genes (violet and green bars in figures 3A, 3B, 3C, 3D, 3G and green bars in 3H and 3I) even though some of them are negatively affected by the doses (red bars in figures 3A, 3B, 3C and 3D, 3G, 3H and 3I).

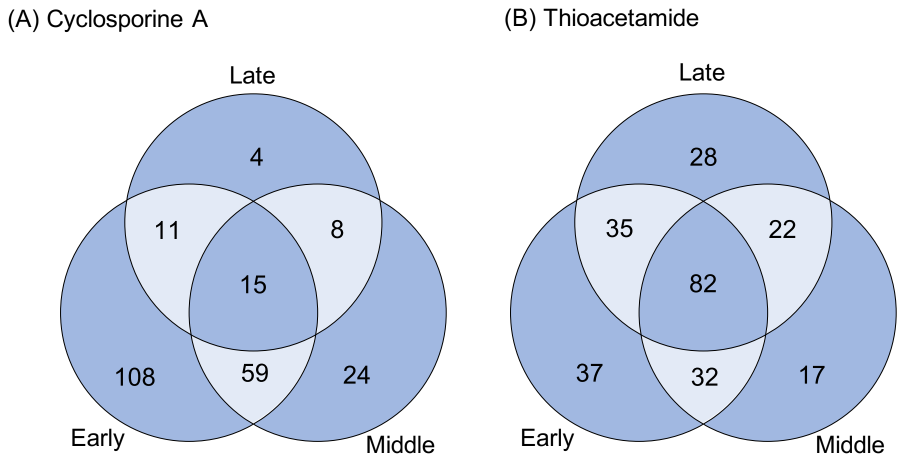

Figure 5. Venn diagram of the number of enriched pathways for each time category (early, middle, late) for the dynamic dose-responsive genes of cyclosporine A (A) and thioacetamide (B).

spond at different dose ranges informs on the sensitivity of certain molecules and regulatory machinery to a specific exposure. The analysis of the dynamic dose-dependent gene profiles might give insights about the harmfulness of a compound. Indeed, substances that activate many genes at low doses are probably more toxic than those exerting resilient responses. This is the case of the two drugs we analyzed, as cyclosporine A shows most of the activation at low and high doses and early and middle time points, while thioacetamide shows most of the activation at low doses at all the time points (Figure 2).

Effect of time and dose on the dynamic MOA

Even though the labels that TinderMIX assigns inform on the gene POD, they do not give insights on the relative impact of the dose and time on the variation of the log-fold-change. In order to dissect these effects and the relative contribution of dose and time to the gene alteration, TinderMIX weighs their effect in the time-dose effect maps (Figure 1B). Indeed, by studying the direction of the gradient in each pixel of the DDRA (Figure 1C), we are able to assess the contribution of time and dose to the dynamic dose-response behaviour of the gene expression, and whether its effect is positive or negative (Figure 1E). Moreover, TinderMIX generates a radar plot that summarizes the relative effect of dose and time onto the DDRG as well as their direction (Figure 3 and 4). There are different scenarios where it is useful to recognize if the genes are more prominently affected by the dose or the time. For example, genes for which the exposure has a predominant effect might be more sensitive to the dose. On the other hand, some genes might be under complex regulatory mechanisms, some of which could be secondary to the exposure itself. Thus, their expression is not only altered in a dose-dependent manner, but also kinetically modulated along the time. Indeed, in both cyclosporine A and thioacetamide, late genes undergo a stronger effect of time than to the dose (Figures 3A, 3B, 3C, 4A, 4B, 4C), as expected since transcriptomic alterations happening 24h after the exposure are more likely to be due to regulatory processes happening downstream

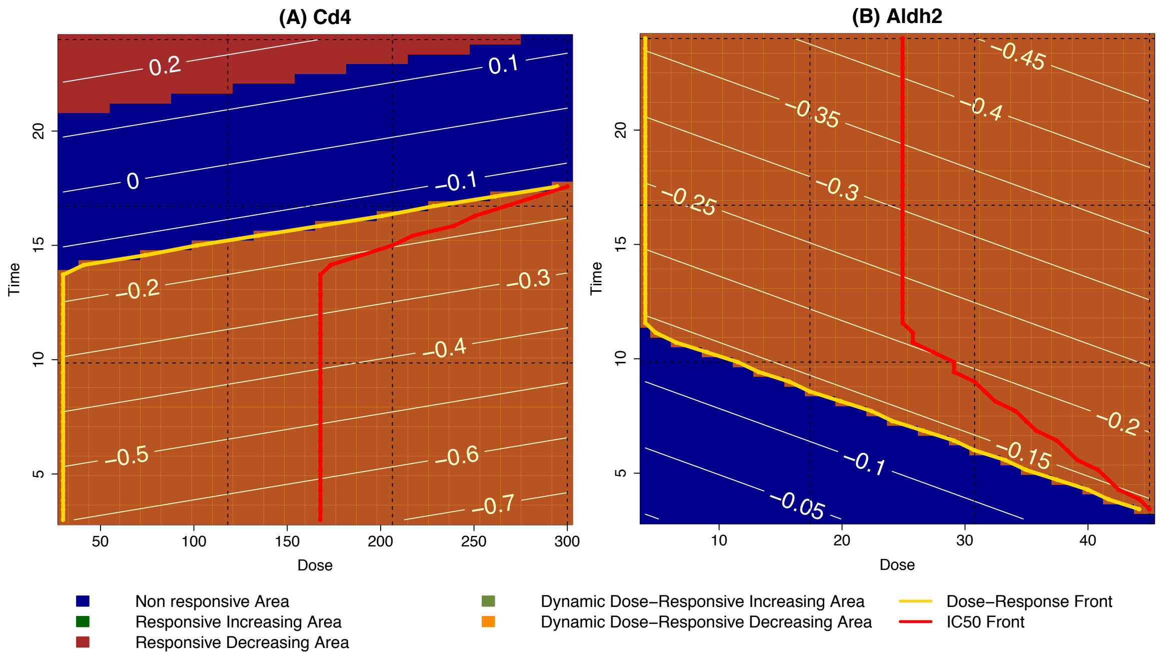

Figure 6. Time-dose effect maps of the Cd4 (A) and Aldh2 (B) genes.

the primary drug induced response. On the other hand, early and middle genes appear to be more affected by the dose, especially in the case of thioacetamide, a compound with a more pronounced known toxic potential (Figures 4D, 4E, 4G, 4H). Therefore, our tool provides better insights about the exposure MOA on a biological system, by providing both quantitative and qualitative estimation of the perturbation with respect to time and dose.

Pathway enrichment analysis

In order to characterize the biological processes underlying the POD labels assigned to the DDRG, we performed KEGG enrichment analysis of the genes belonging to the nine label categories previously identified (additional files 4 and 5). We further grouped the enriched pathways based on the time of activation to draw a kinetic map of the events in the exposure time (Figure 5). Cyclosporine A is a known immunosuppressant drug, with a low toxic potential. Indeed, at early time points, 108 deregulated pathways were found (Figure 5A) while, at middle and late time points, only few deregulated pathways (24 and 4 respectively) were obtained. Cyclosporine A is known to inhibit the activation of T lymphocytes by blocking the activity of calcineurin phosphatase [22]. In fact, several interleukins and chemokine-driven immune system mechanisms were found significantly deregulated at early time points. In particular, Th1 and Th2 cell differentiation pathways were found altered. Among the main effectors of such pathways Cd4, one of the main markers of T lymphocytes, was labelled by TinderMIX as a sensitive-early DDR gene. On the other hand, thioacetamide exposure has been associated with liver toxicity and inflammatory cells infiltration [23]. As it might be expected for a more toxic drug, more deregulated pathways at any time points were retrieved in our analysis (Figure 5B). Among the ones enriched at early time points, infectious disease pathways such as Hepatitis B and C, as well as apoptosis pathway, were enriched and both apoptotic and necrosis related genes were up/down-regulated, such as Fadd, Fas, Bad and Bid. Consistently with an hepatotoxic induced effect, the Aldh2 gene, which is known to be altered in patients with chronic hepatitis and non-alcoholic cirrhosis, was found also deregulated in intermediate pathways [24].

Visual inspection of the gene maps

To complement the information given by the POD labels, the time-dose effect maps of the DDRG allow to visualize the whole kinetics of the log-fold-changes in the joint dose-time space. Furthermore the levels of the contour plots specify if the gene is up (+) or down (-) regulated. The effect maps of the previously identified genes are described as an example. As we can see from figure (Figure 6A), Cd4 is marked as sensitive-early, and it is down-regulated at early time points (log-fold-change in [-0.7,-0.4]). Furthermore, the log-fold-change increases at middle time points (log-FC in [-0.4,-0.1]) and is eventually upregulated at late time points (log-FC in [0.1,0.2]). The expression pattern of this gene changes over time in concordance with the known MOA of cyclosporine A[22]. On the other hand, the Aldh2 gene is labelled as sensitive-middle since the DDRA begins at the lowest doses and middle time points (Figure 6B). It does not show any activity at early time points with low and middle doses, while it is dynamically dose-dependent and down-regulated already at early time points with high doses. Its strength of downregulation increases with both time and dose (log-fold-change in [-0.15,-0.45]). The overall downregulation visible in the map has already been reported in previous studies demonstrating that, in rats, thioacetamide can directly inhibit the Aldh2 isoenzyme[25].

Conclusion

In this study, we proposed TinderMIX, a novel methodology for the joint time and dose modelling of toxicogenomics data. TinderMIX consists of different steps that combine polynomial model fitting, active region extraction and pathways enrichment analysis in order to identify genes with joined dose and time patterns of responsiveness. TinderMIX allows the user to study the dynamic behaviour of the genes in the joint dose-time space, by interpolating the omics feature levels and filling the gaps between the doses and time points tested in the experiment. TinderMIX automatically assigns to the responsive genes an activation label that specifies the joint dose and time point of departure (POD) of the gene and estimates the strength of the effect of time and dose on each gene activation. Moreover, it allows to graphically inspect the gene maps as contour plots. Each gene map can give insights into the dynamic and dose-dependent shape of the gene log-fold-change. It easily shows the point of departure of the gene and it highlights the monotonic trend of the responsive area. The TinderMIX methodology also helps in grouping the genes based on the activation label and to identify the set of pathways associated with each group in order to better characterise the underlying biological mechanisms. In conclusion, TinderMIX can be used to investigate the point of departure of genes with respect to dose and time point upon chemical exposure with an integrated analytical approach.

Availability of source code and requirements

The TinderMIX method is available in the form of an R package, which is available via a git repository:

- Project name: TinderMIX
- Project home page: https://github.com/grecolab/TinderMIX
- Operating system(s): Platform independent
- Programming language: R
- Other requirements: Java
- License: GNU GPL (>= 3)

Availability of supporting data and materials

The data used to showcase the TinderMIX methodology are available in the git repository at https://github.com/grecolab/TinderMIX/tree/master/sample_data.

Additional Files

- Additional file 1: TinderMIX pseudo-code
- Additional file 2: list of dynamic dose-responsive genes for cyclosporine A
- Additional file 3: list of dynamic dose-responsive genes for thioacetamide
- Additional file 4: list of pathways for cyclosporine A
- Additional file 5: list of pathways for thioacetamide

List of abbreviations

- ANOVA: analysis of variance
- BMD: benchmark dose analysis
- DDR: dynamic dose response
- DDRA: dynamic dose responsive area

- DDRG: dynamic dose responsive gene
- IC50: half maximal inhibitory concentration
- KEGG: Kyoto Encyclopedia of Genes and Genomes
- log-FC: logarithm of fold-change
- MOA: mechanism of action
- NOAEL: no-observed-adverse-effect-level
- Open TG-GATEs: Open Toxicogenomics Project-Genomics Assisted Toxicity Evaluation System
- POD: point of departure
- TinderMIX: Time-Dose INtegrated moDelling of Omics data

Declarations

Competing Interests

The author(s) declare that they have no competing interests.

Funding

This study was supported by the Academy of Finland [grant number 322761] and the EU H2020 NanoSolveIT project [grant number 814572].

Author's Contributions

Conceptualization: A.S., D.G., L.A.S., Data curation: A.S., Formal Analysis: A.S., Funding acquisition: D.G., Methodology: A.S., M.F., M.P., Project administration: D.G., A.S., Software: A.S., M.F., M.P., Supervision: D.G., Visualization: A.S., Writing – original draft: A.S., M.F., G.d.G., A.F., D.G. Writing - review and editing: A.S., M.F., G.d.G., A.F., L.A.S., M.P., D.G. All authors have read and agreed to the published version of the manuscript.

References

1. Authority EFS, Aguilera J, Aguilera-Gomez M, Barrucci F, Cocconcelli PS, Davies H, et al. EFSA Scientific Colloquium 24-'omics in risk assessment: state of the art and next steps. EFSA Supporting Publications 2018;15(11):1512E.
2. Aardema MJ, MacGregor JT. Toxicology and genetic toxicology in the new era of "toxicogenomics": impact of "-omics" technologies. In: Toxicogenomics Springer; 2003.p. 171–193.
3. Shi L, Campbell G, Jones WD, Campagne F, Wen Z, Walker SJ, et al. The MicroArray Quality Control (MAQC)-II study of common practices for the development and validation of microarray-based predictive models. Nature biotechnology 2010;28(8):827.
4. Yasokawa D, Iwahashi H. Toxicogenomics using yeast DNA microarrays. Journal of bioscience and bioengineering 2010;110(5):511–522.
5. Lettieri T. Recent applications of DNA microarray technology to toxicology and ecotoxicology. Environmental health perspectives 2006;114(1):4–9.
6. Davis AP, Grondin CJ, Johnson RJ, Sciaky D, King BL, McMorran R, et al. The comparative toxicogenomics database: update 2017. Nucleic acids research 2017;45(D1):D972–D978.
7. Igarashi Y, Nakatsu N, Yamashita T, Ono A, Ohno Y, Urushidani T, et al. Open TG-GATEs: a large-scale toxicogenomics database. Nucleic acids research 2015;43(D1):D921–D927.
8. Thomas RS, Allen BC, Nong A, Yang L, Bermudez E, Clewell III HJ, et al. A method to integrate benchmark dose estimates with genomic data to assess the functional effects of chemical exposure. Toxicological Sciences 2007;98(1):240–248.
9. Pagé-Larivière F, Crump D, O'Brien JM. Transcriptomic points-of-departure from short-term exposure studies are protective of chronic effects for fish exposed to estrogenic chemicals. Toxicology and applied pharmacology 2019;378:114634.
10. Labib S, Williams A, Yauk CL, Nikota JK, Wallin H, Vogel U, et al. Nano-risk Science: application of toxicogenomics in an adverse outcome pathway framework for risk assessment of multi-walled carbon nanotubes. Particle and fibre toxicology 2015;13(1):15.
11. Dorato MA, Engelhardt JA. The no-observed-adverse-effect-level in drug safety evaluations: use, issues, and definition (s). Regulatory toxicology and pharmacology 2005;42(3):265–274.
12. Serra A, Saarimäki LA, Fratello M, Marwah VS, Greco D. BMDx: a graphical Shiny application to perform Benchmark Dose analysis for transcriptomics data. Bioinformatics;.
13. Yang L, Allen BC, Thomas RS. BMDExpress: a software tool for the benchmark dose analyses of genomic data. BMC genomics 2007;8(1):387.
14. Kuo B, Francina Webster A, Thomas RS, Yauk CL. BMDExpress Data Viewer-a visualization tool to analyze BMDExpress datasets. Journal of Applied Toxicology 2016;36(8):1048–1059.
15. Albrecht M, Stichel D, Müller B, Merkle R, Sticht C, Gretz N, et al. TTCA: an R package for the identification of differentially expressed genes in time course microarray data. BMC bioinformatics 2017;18(1):33.
16. Nueda MJ, Tarazona S, Conesa A. Next maSigPro: updating maSigPro bioconductor package for RNA-seq time series. Bioinformatics 2014;30(18):2598–2602.
17. Schüttler A, Altenburger R, Ammar M, Bader-Blukott M, Jakobs G, Knapp J, et al. Map and model—moving from observation to prediction in toxicogenomics. GigaScience 2019;8(6):giz057.
18. Teräsvirta T, Mellin I. Model selection criteria and model selection tests in regression models. Scandinavian Journal of Statistics 1986;p. 159–171.
19. Team RC, et al. R: A language and environment for statistical computing 2013;.
20. Irizarry RA, Bolstad BM, Collin F, Cope LM, Hobbs B, Speed TP. Summaries of Affymetrix GeneChip probe level data. Nucleic acids research 2003;31(4):e15–e15.
21. Leek JT, Johnson W, Parker H, Jaffe A, Storey J. sva: Surrogate Variable Analysis R package version 3.10. 0. DOI 2014;10:B9.
22. Matsuda S, Koyasu S. Mechanisms of action of cyclosporine. Immunopharmacology 2000;47(2-3):119–125.
23. Schyman P, Printz RL, Estes SK, Boyd KL, Shiota M, Wallqvist A. Identification of the toxicity pathways associated with thioacetamide-induced injuries in rat liver and kidney. Frontiers in pharmacology 2018;9:1272.
24. Oniki K, Morita K, Watanabe T, Kajiwara A, Otake K, Nakagawa K, et al. The longitudinal effect of the aldehyde dehydrogenase 2* 2 allele on the risk for nonalcoholic fatty liver disease. Nutrition & diabetes 2016;6(5):e210–e210.
25. Song BJ, Abdelmegeed MA, Yoo SH, Kim BJ, Jo SA, Jo I, et al. Post-translational modifications of mitochondrial aldehyde dehydrogenase and biomedical implications. Journal of proteomics 2011;74(12):2691–2702.

supplementary file 1

Click here to access/download
Supplementary Material
S1_TinderMIX_pipeline.docx

supplementary file 2

Click here to access/download
Supplementary Material
S2_CyclosporineA_genes_all_info.xlsx

Click here to access/download
Supplementary Material
S3_thioacetamide_genes_all_info.xlsx

supplementary file 4

Click here to access/download
Supplementary Material
S4_cyclosporineA_kegg.xlsx

supplementary file 5

Click here to access/download
Supplementary Material
S5_thioacetamide_kegg.xlsx

Click here to download Personal Cover
Cover_Letter_GigaScience.docx

Tampere University

To the Editor of *GigaScience*

Dear Editor,

Please find enclosed the manuscript entitled **"TinderMIX: Time-Dose INtegrated moDelling of toxicogenomics data"** by Angela Serra, Michele Fratello, Giusy del Giudice, Laura Aliisa Saarimäki, Michelangelo Paci, Antonio Federico, and Dario Greco, to be considered for publication in *GigaScience.*

Toxicogenomics approaches have been increasingly employed to resolve the molecular mechanism of action of chemicals. Most of the toxicogenomics experiments consist in testing substances at multiple doses and time points. One of the main challenges consists in identifying the molecular alteration patterns that are correlated to both dose and time. Existing analytical methods allow to study the dose dependency one temporal point at a time, thus they are not able to identify dynamic points of departure.

Here we propose TinderMIX, a novel computational approach for toxicogenomics data modelling that simultaneously analyzes the effect of time and dose. Furthermore, TinderMIX estimates a joint time and dose point of departure for each gene with dynamic dose response activity. Our tool is available in the form of an R package via our github page: https://github.com/grecolab/TinderMIX. The data used in the manuscript to showcase the TinderMIX methodology and the script needed to replicate our analysis.

We believe that our study will be of broad interest for the readership of *GigaScience* and has potential implications for the development of new chemical safety assessment methods. All co-authors have approved the manuscript and its submission to *GigaScience* and none of the findings have been published elsewhere. The authors have no conflicts of interest to declare.

As reviewers, we would like to suggest:

1. Antreas Afantitis, Novamechanics, afantitis@novamechanics.com
2. Haralambos Sarimveis, National Technical University of Athens, hsarimv@central.ntua.gr
3. Olivier Joubert, Institut Jean Lamour, CNRS 7198, University of Lorraine, olivier.joubert@univ-lorraine.fr

Sincerely,

Dario Greco
PhD, Associate Professor, Academy of Finland Research Fellow

Faculty of Medicine and Life Sciences
Tampere University
Finland
