# Supplementary material for: TinderMIX: Time-dose integrated modelling of toxicogenomics data
Source: Gigascience. 2020 May 25;9(5):giaa055. doi: 10.1093/gigascience/giaa055 (PMC7247400; doi:10.1093/gigascience/giaa055)
Supplement: giaa055_Supplemental_Files [file giaa055_supplemental_files.zip › S1_TinderMIX_pipeline.pdf]

Algorithm 1: TinderMIX pipeline

INPUT: geneMap, activityThreshold, maxDose
OUTPUT: dose response area, dose response front, IC50 front

IF max(abs(geneMap)) < activityThreshold
    Non active gene

Identify eligible and non eligible regions of geneMap depending on the activityThreshold
eligible: abs(geneMap) >= activityThreshold
nonEligible: abs(geneMap) < activityThreshold

Identify increasing and decreasing eligible area with respect to the dose
Compute the matrices Gd and Gt of gradients dose and time components respectively, in the eligible region

Partition the eligible region in increasing or decreasing with respect to the dose component of the gradients

eligibleIncreasing: Gd >= 0
eligibleDecreasing: Gd < 0

IF none of the two regions (eligibleIncreasing, eligibleDecreasing) include maxDose
    Non responsive gene

Identify candidate area
IF eligibleIncreasing includes maxDose and eligibleDecreasing does not include maxDose
    candidateRegion = eligibleIncreasing
IF eligibleIncreasing does not include maxDose and eligibleDecreasing includes maxDose
    candidateRegion = eligibleDecreasing

IF both eligibleIncreasing and eligibleDecreasing include maxDose then compute:
    areaInc, areaDec: eligibleIncreasing and eligibleDecreasing area
    maxTpInc, maxTpDec: number of timepoints in eligibleIncreasing and eligibleDecreasing areas reaching maxDose
    mDoInc, mDoDec: minimum dose included in Compute eligibleIncreasing and eligibleDecreasing area

scoreInc = areaInc + maxToInc - mDoInc
scoreDec = areaDec + maxToDec - mDoDec

IF scoreInc > scoreDec
    candidateRegion = eligibleIncreasing
ELSE
    candidateRegion = eligibleDecreasing

doseResponseArea = candidateRegion

filter rows from doseResponseArea
For each row_i in the geneMap:
    IF row_i contains portion of the doseResponseArea that do not include maxDose
        Remove row_i from the doseResponseArea
    IF row_i contains portion of eligibleIncreasing and eligibleDecreasing region
        Remove row_i from the doseResponseArea

Identify the dose-response front, by connecting the point identified as the smallest dose in for each time point in the doseResponseArea

Identify the IC50 front by connecting all the points identified as the dose that gives 50% of response for each time point in the doseResponseArea

Assign a label to each gene
Split the geneMap in a 3 by 3 grid, and label the grid as early, middle and late on the time point side, and sensitive, intermediate and resilient on the dose side.

Identify the cell of the grid that contains the portion of the doseResponseArea with the lowest dose and the lowest time point.
