# Supplementary figures and images for: TinderMIX: Time-dose integrated modelling of toxicogenomics data
Source: Gigascience. 2020 May 25;9(5):giaa055. doi: 10.1093/gigascience/giaa055 (PMC7247400; doi:10.1093/gigascience/giaa055)

(A) Cyclosporine A

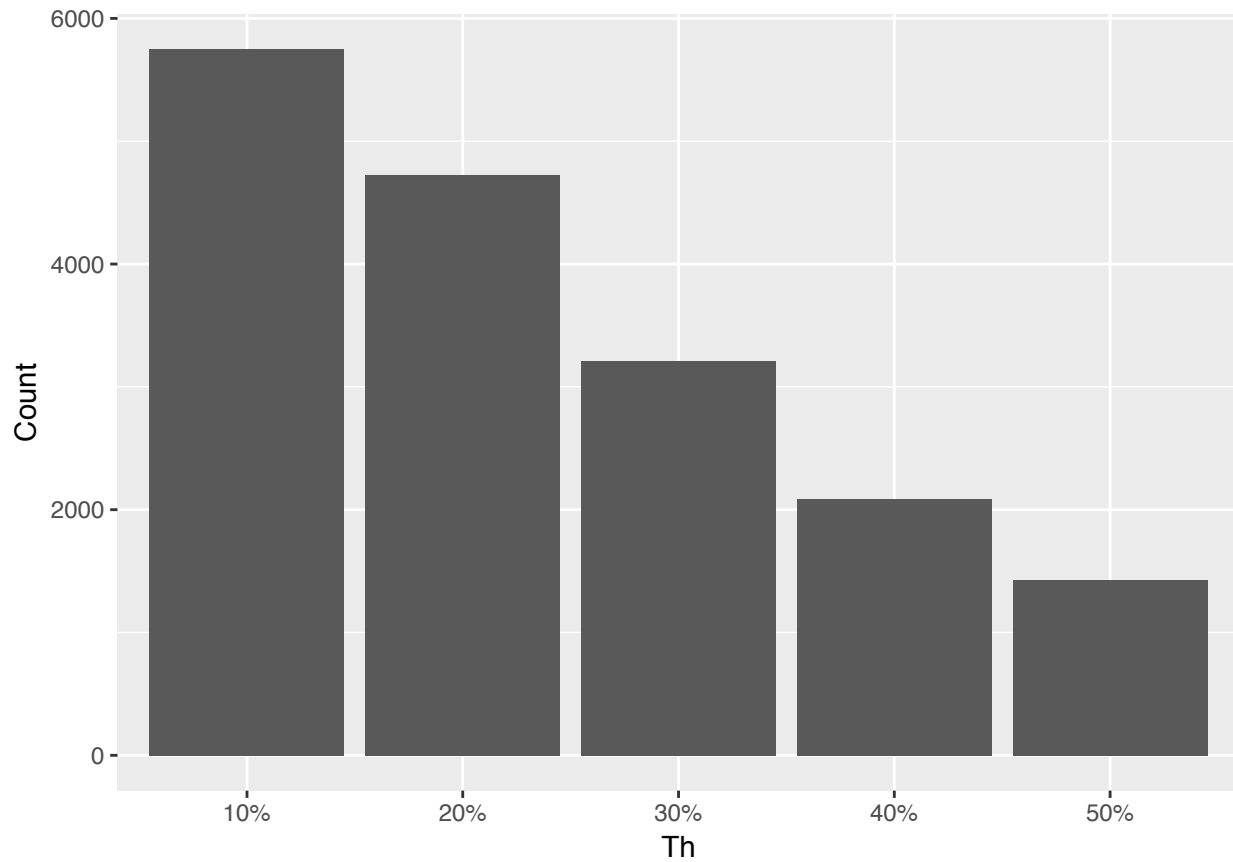

(B) Thioacetamide

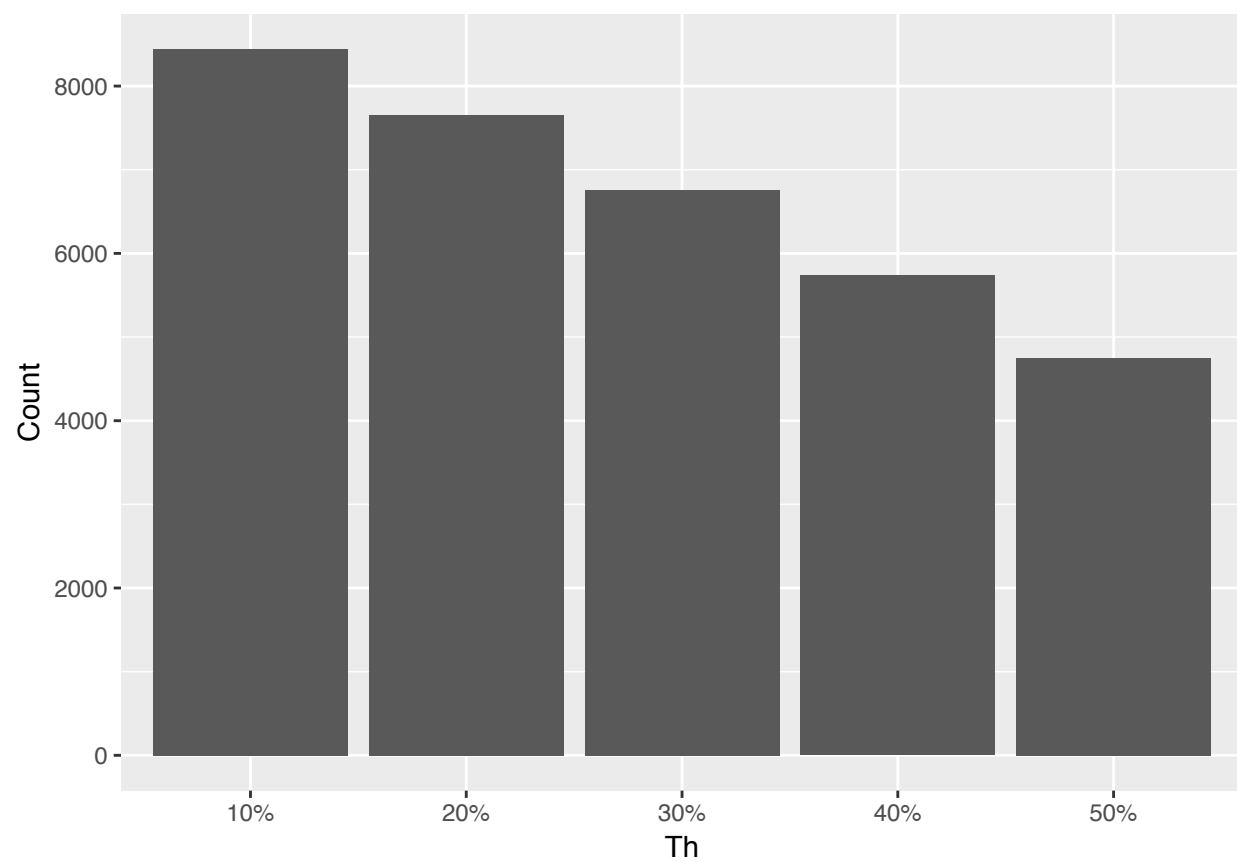

Supplement: giaa055_Supplemental_Files [file giaa055_supplemental_files.zip › S4_thresholds.pdf]
