# Supplementary material for: TinderMIX: Time-dose integrated modelling of toxicogenomics data
Source: Gigascience. 2020 May 25;9(5):giaa055. doi: 10.1093/gigascience/giaa055 (PMC7247400; doi:10.1093/gigascience/giaa055)
Supplement: giaa055_Response_to_Reviewer_Comments_Original_Submission [file giaa055_response_to_reviewer_comments_original_submission.pdf]

Author's Response To Reviewer Comments

Close

Manuscript ID: GIGA-D-20-0005

Title: Time-Dose INtegrated moDelling of toxicogenomics data

Dear editor,
please find enclosed a revised version of our manuscript. We addressed the comments raised by the reviewers. We would like to thank the reviewers for constructive feedback. We believe that this revised manuscript is significantly improved from the initial submission and we look forward to your positive feedback.

Yours Sincerely,
Dario Greco
PhD, Associate Professor,
Faculty of Medicine and Health Technology
Tampere University Finland

____

Reviewer 1

General :
In this paper, the authors propose a new approach, called TinderMIX, for simultaneously studying time and dose effects in toxicogenomics. Although many toxicogenomics experiments include data from different doses measured at different time points, only a few bioinformatics approaches to integrate time and dose effects exist, and their applications are limited to network identification and response modeling. The new method proposed by the authors extends existing methods by identifying genes with joined dose and time patterns of responsiveness, and predicting the molecular alteration values for the doses and time-points not included in the original experiment. Moreover, the approach assigns an activation label to each gene describing the time (early, middle, late) and dose effect (sensitive, intermediate, resilient). Genes are grouped based on the activation label, and pathway analysis can be performed on each group in order to better characterise the underlying biological mechanisms. Moreover, the method is available as a user-friendly R package on GitHub.
To the best of my knowledge, this is the first bioinformatics approach performing such an extensive analysis of both time and dose effects in toxicogenomics. This makes the method described in this manuscript of particular interest for improving our understanding of the mode of action of compounds. However, before I can recommend the manuscript for publication in GigaScience, a few issues have to be addressed:

Major :
* Materials and methods, section Time-dose Fitting: the authors explain that they use a desired activity threshold corresponding to a 10% increase/decrease with respect to controls. However, the authors have to explain why the threshold has been set to 10% and not to a higher or lower value.

We thank the reviewer for the useful remark. The threshold of 10% is the default threshold used in the literature for BMD analysis of transcriptomic data, as already published by Thomas et al., Yang et al., Serra et al.

Thomas, R.S., Allen, B.C., Nong, A., et al. 2007. A method to integrate benchmark dose estimates with genomic data to assess the functional effects of chemical exposure. Toxicological Sciences 98(1), pp. 240–248.
Yang, L., Allen, B. C., & Thomas, R. S. (2007). BMDExpress: a software tool for the benchmark dose analyses of genomic data. BMC genomics, 8(1), 387.
Serra, A., Saarimäki, L. A., Fratello, M., Marwah, V. S., & Greco, D. BMDx: a graphical Shiny application

to perform Benchmark Dose analysis for transcriptomics data. Bioinformatics.

We added the following sentence in the “Time-dose effect map prediction and Dynamic dose-responsive genes identification” paragraph:

“The selected threshold of 10% is a default threshold used in BMD analysis of transcriptomics data [Thomas, 2007; Yang 2007; Serra 2020].”

Moreover, the authors need to explain how the value of the threshold influences the results of their analysis. If the authors would have chosen a slightly lower or higher threshold, how much would their results differ from the current results?

We thank the reviewer for the useful suggestion. We investigated the behaviour or TinderMIX with the following thresholds: 10%, 20%, 20%, 40% and 50%. We analysed the number of dynamic-dose-responsive genes and their labels identified at each threshold. In both the two drugs present in our case study, the number of dynamic-dose-responsive genes linearly decreases with the increasing of the thresholds.
We added the following figures as supplementary materials and the following text at the end of paragraph “Dynamic dose-dependent mechanism of action (MOA)”.

“Furthermore, the sensitivity of the TinderMIX method to the activation threshold, was investigated. The analyses were run for different activation thresholds (10\%, 20\%, 30\%, 40\% and 50\%). In both the two drugs present in our case study, the number of dynamic-dose-responsive genes decreases with the increasing of the thresholds.”

Number of dynamic-dose responsive genes in cyclosporine A (A) and thioacetamide (B) at different activation thresholds.

* Caption Figure 3: "Considering the dose, the log-fold-change of sensitive-middle and sensitive-late genes mainly decreases as the dose increases." I agree with the authors that this is the case for the sensitive-late genes. However, for the sensitive-middle genes, I do not understand this conclusion. Looking at the figure for the sensitive-middle genes, I observe that the colored area in the right quadrants, displaying an increase in log-fold-change with increasing dose is larger than the area in the left quadrants, displaying a decrease. In particular, the purple bar is much larger than the red bar.

We thank the reviewer for pointing this out. We removed sensitive-middle from the sentence.

Minor :
* Abstract : The authors claim that 'Existing methods for toxicogenomics data analysis allow studying molecular alteration after the exposure (or treatment) at each time point individually.' Although this is the case for the majority of the methods, there exist already a few methods for integrating the effects of time and dose, that consider the whole time profile instead of looking at each time point individually (see e.g. PMID: 31409281, PMID: 28032149, PMID: 31140561 (reference already mentioned by the authors in the introduction)). I therefore propose to replace "Existing methods …" by "The majority of existing methods …".

We thank the reviewer and we amended the text accordingly.

* Figure 1E: Explain symbols D+, D-, d+, d-, T+, T-, t+, t- in the figure caption, as you refer to the figure before explaining these symbols in the text.

We agree with the reviewer and we added to the figure caption the following text:

“The letters d and t stand for dose and time, respectively. In the label, a capital letter means that there is a stronger effect of a variable with respect to the other. If the two letters are both capitals it means that the effect is of similar intensity. Concordance in the increase of the fold change and dose/time is indicated with a +, while decrease with a -.”

* Materials and methods, section Enrichment of biological pathways: "KEGG enrichment analysis is

performed by using the methodology implemented in the FunMappOne tool ??." Replace "??" by the reference to the paper on the FunMappOne tool.

We apologize for the missing reference and we added it.

* Materials and methods, section Dataset collection: "For every drug pathological score was computed by …" -> "Every drug pathological score was computed by …"

We amended the text accordingly to the reviewer suggestion

* Materials and methods, section Dataset collection: "Cyclosporine A and thioacetamide were selected as representative candidates for drugs with low and high toxic impact, respectively." Add literature references that explain that Cyclosporine A is a drug with low toxic impact and thioacetamide a drug with high toxic impact.

By following the reviewer suggestions we added the following references for Cyclosporine A and Thioacetamide:

Ragab, A. R., Al-Mazroua, M. K., & Al-Dakrory, S. A. (2013). Cyclosporine toxicity and toxicokinetics profiles in renal transplant recipients. J Clinic Toxicol, 3(154), 2161-0495.
Husein, M., & Pingle, S. (1992, October). Effect of cyclosporine A at therapeutic and toxic doses on the superluteinized ovaries in BALB/c mice. In Transplantation proceedings (Vol. 24, No. 5, pp. 1663-1668).

Lin, You-Yen, et al. "Thioacetamide-induced liver damage and thrombocytopenia is associated with induction of antiplatelet autoantibody in mice." Scientific reports 9.1 (2019): 1-11.
Alomar, Mohammed Yahya, and Atef Mohammed Al-Attar. "Effect of basil leaves extract on liver fibrosis induced by thioacetamide in male rats." Int. J. Pharmacol 15 (2019): 478-485.

* Materials and methods, section Dataset collection: add link to website and literature reference for the Open TG-Gates dataset

We added the reference and link to the website for the Open TG-Gates dataset

* Materials and methods, section Dataset collection: "12 are replicates for each dose level". Are these biological or technical replicates? Please explain already in this paragraph that you use the in vivo data.

We clarified in the manuscript that the data come from in vivo rat liver tissue and that they are biological replicates.

* Caption Table 1: "Linear is the number of DDR that is fitted by a liner, poly2 and poly3 are the numbers of DDR genes that are fitted by a linear, 2nd and 3rd order polynomial function respectively." -> "Linear, Poly2 and Poly3 are the numbers of DDR genes that are fitted by a linear, 2nd and 3rd order polynomial function respectively."

We amended the table caption

* Caption Figure 3 refers to Figures 2A - 2I. Has to be 3A-3I? Please label the figures with A, B, C, …, I. Also in the caption of Figure 3: "the effect of time is more evenly distributed across the possible combinations (Figure 2)." However, this information is not shown in Figure 2. Please refer to the correct figure.

We thank the reviewer for the useful comment and we added the missing labels to both figure 3 and 4.

* Additional files 2 and 3: I suggest to explain the abbreviations in the column names of the Excel tables in the description of the additional files.

We acknowledge that the supplementary files 2 and 3 were a little bit messy. We removed the redundant columns and we added a description of the column names in the description of the additional files as follows:

“The file contains the following information: (1) dose_time_comparison: specifies if the activation is

more dependent on the dose or the time; (2) Gene Description: is a text description of the gene; (3) Gene Symbol; (4) Joint Label: is the POD label; (5) gene_sign: specifies if the gene activation is increasing or decreasing with respect to the dose; (6) MeanFC: mean log-fold-change of the gene in the POD area; (7) adj.pval: pvalue of the fittied polynomial model;"

* Caption Figure 4 refers to Figures 3A - 3I. Has to be 4A-4I? Please label the figures with A, B, C, …, I.

We added the missing labels to both figure 3 and 4.

* Caption Figure 4: "direct correlation between the increase of the fold-change and the dose": authors refer to the purple and green bars. However, there are two types of green in the figures. Do you mean the blue green bars?

We thank the reviewer for pointing this out. Colors are only related to the position of the bars in the four quadrants. In this particular case, we refer only to the bars that are on the right side of the Y axis.

* The terms "Dynamic Dose−Responsive Increasing Area" and "Dynamic Dose−Responsive Decreasing Area" only appear in the caption of Figure 6. It would be more clear if these terms were already explained in the overview of the methods (Figure 1c + corresponding text).

We thank the reviewer and we modified the caption of Figure 1 in order to better describe the increasing and decreasing behaviour of the dynamic dose responsive areas.

* Conclusion: "pathways enrichment analysis" -> "pathway enrichment analysis".

We corrected the typo

Reviewer 2

The paper introduces a new toxicogenomics data modelling approach which considers simultaneously the effect of both time and dose in the calculation of points of departure. The method is applied on individual genes, but using enrichment analysis it is generalized and extended to the level of pathways and mechanisms of action.

The proposed method is novel, very interesting to the readers of GigaScience and paves the way for new developments in the area of chemicals safety and risk assessment by considering dose and time simultaneously. Therefore, I am happy to recommend the acceptance of the paper.

I have some minor comments that may contribute to further improve the manuscript and the presentation of the methodology:

1) The selected linear regression models include 1st , 2nd and 3rd order polynomials. These are not the usual models used in the standard BMD method (Gamma, Logistic models etc.). Is there any particular reason for this choice?

We thank the reviewer for raising this commnet. We would like to point out that polynomials models are used in classical BMD analysis of transcriptomics data, as already indicated in previous studies:

Thomas, R.S., Allen, B.C., Nong, A., et al. 2007. A method to integrate benchmark dose estimates with genomic data to assess the functional effects of chemical exposure. Toxicological Sciences 98(1), pp. 240–248.
Yang, L., Allen, B. C., & Thomas, R. S. (2007). BMDExpress: a software tool for the benchmark dose analyses of genomic data. BMC genomics, 8(1), 387.
Serra, A., Saarimäki, L. A., Fratello, M., Marwah, V. S., & Greco, D. BMDx: a graphical Shiny application to perform Benchmark Dose analysis for transcriptomics data. Bioinformatics.

The main idea of this work is to propose a methodology that jointly models time and dose to highlight the importance of the dynamics in the POD identification. Even though polynomial functions are not the only models used in BMD analysis, they can be used for reliable approximation of other functions.

Differently from the polynomials, the gamma and logistic models do not have a straightforward or unique generalization to more than one input variables. This would require a deeper evaluation of their applicability in the integrated time-dose modelling of gene expression data that might be done in further studies.

“These models are selected as representative of those used in classical BMD analysis [Thomas, 2007; Yang, 2007; Serra, 2000]. Indeed, unlike the gamma and logistic models, polynomials are easily generalised to more than one input variables and they can be used for reliable approximation of other functions.”

2) It is mentioned in p. 3 that the desired activity threshold is a 10% increase or decrease with respect to controls. How this is compared with Fig. 1 where the threshold seems to be higher than 10%?

We first want to clarify that the maps in figure 1 are a mock-up used to explain how to read and interpret them, but they are not calculated on real data.
Moreover, a difference in the expression of the treatment vs the controls of at least 10% corresponds to a fold-change of at least 1.1 that equals a log2FC of ~0.13.
In figures 1C-1D, the first log2FC value above the threshold is slightly less than 0.15, which corresponds to a fold-change of 2^0.15 = ~1.11. This corresponds to an increase of ~11%, which is above the defined threshold.

3) What should be the minimum percentage of a region in the 3x3 grid belonging to the green (or red) area that defines it as a POD? For example, if a very small part of the sensitive-early region was green, it is still enough to specify it as a POD?

We thank the reviewer for raising this important point.

In the analysis shown as the case study of our manuscript, we decided to focus on PODs at the lowest dose and earliest time of exposure. This is in line with a general reasoning in toxicology, where we are interested in identifying the PODs based on the absolute sensitivity of the biological system (in time and dose).
However, we acknowledge that there are some marginal cases where the POD area that we identify can have a small coverage, but we have seen that this happens rarely and the labelling strategy works pretty well. Also, It is important to note, that these labels are meant to give an indication of the POD and help in grouping the genes and give an interpretation but they are not self explanatory of the all gene expression dynamic.

Nonetheless, we implemented multiple options to assign labels to the genes in the TinderMIX library. In particular, we offer the following alternative strategies:
Most left: a gene is labelled with the labe of the region containing the lowest dose and early time point of the dynamic-dose-responsive area
presence: a gene is labeled with all the labels of the regions covered by the dynamic-dose-responsive area.
Cumulative: a gene is labeled with the labels of the regions needed to cover x% of the border of the dynamic-dose-responsive area. X is a threshold given in input by the user
Mix: for each gene, a combined score is created for each region of the 3x3 grid. The score takes into account the proximity with the (0,0) coordinate, the coverage of the area and the number of points of the border of the dynamic-dose-responsive area contained in the region. Finally the gene is labeled according to the region with the higher score.

We have added a new Figure 2 in the manuscript, describing the different labeling strategies and a “POD label assignment” subsection in the Materials and Method section that contains the following text:

“The dose-time space is partitioned into 3X3 regions, where each dose can be labelled as sensitive, intermediate or resilient, and each time point is labelled as early, middle or late. Different strategies to identify the POD of the dynamic-dose-responsive genes are implemented in TinderMix. The first method is called "most left" strategy. It looks for the first available POD region by identifying the most sensitive point of activation (lowest dose) and then its corresponding earliest time point. The second strategy, called "presence", identifies as PODs all the regions containing the DDRA border marked as a yellow line (Figure \ref{fig:method}). The third method, called “cumulative”, ranks the regions in the 3X3 scheme based on the percentage of points of the border of the DDRA that they contain. Then the regions needed to cover the X\% of the border are identified and marked as POD. X is a threshold identified by the user.

In case of X = 100, the cumulative method gives the same result of the presence method. The fourth method, called "mix", creates a score for each region that takes into account how close the region is to the lowest dose and earliest time point, how much area of the region is covered by the DDRA and how many points of the DDRA border are contained in it. The region with the higher score is selected as POD. When the most sensitive and most early region is also the one with higher coverage, this method and the most left one give the same results.
In all the approaches the final POD label is obtained by concatenating the dose and time of the single labels. For example, a gene with label sensitive-early is a gene that shows a response already at low doses and early time points. On the other hand, a resilient-late gene shows a response only at high doses and late time points.
It is important to highlight that the labeling strategies are meant to give an indication of the POD, but they are not self explanatory of the whole gene expression dynamic.
The presence and cumulative approaches identify multiple regions as POD giving a better approximation of the shape of the DDRA area. These two approaches are suggested when the focus of the study is on a few genes. On the other hand, the most left and mix approaches identify only the starting point of the gene activation and can be easily used to group genes and give biological interpretation of chain of events. In the analyses conducted in this work we have used the most left approach because it follows the toxicological assumption that a toxicant is considered active at the lowest dose and earliest time point at which its effect is significantly deviating from the control status"

Different methodologies implemented to identify the dynamic-dose-response POD on a sample gene map. With the "most left" method (A) the POD area is marked as Sensitive-Early, since the activation DDRA is already present at the lowest dose and shortest time-point. With the "cumulative" method (B), the POD is marked as Sensitive-Early, Sensitive-Middle, and Sensitive-Late. The label is obtained by using 80% of the cumulative length of the DDRA border (yellow line). With the "presence" method (C) the POD is labeled as Sensitive-Early, Sensitive-Middle, Sensitive-Late, Intermediate-Early, and Resilient-Early. With the "mix" method (D) the POD is labeled as Sensitive-Middle since the DDRA starts already at lower doses and shorter time points and the area is also the one with a higher coverage by the DDRA.

4) On the POD selection again, could there be a nonlinear case where the early-sensitive region is completely blue, but the early-intermediate and middle-sensitive are green? In this case, which region is selected as the POD?

The way in which we implemented the most left labelling strategy is that we identify the smallest possible dose that is contained in the dynamic-dose-responsive area. Then, for that dose we identify the earliest time point. So in the case highlighted by the reviewer, the label would be assigned as sensitive-middle.

Some typos:
Figure 1 caption: dow -> down

We corrected the typo

p. 6 Two question marks under "Enrichment of biological pathways"

That was a missing reference to funmappone that we now added to the manuscript.

p. 7 Caption of Table 1 is not syntactically correct.

We rephrased the caption of table 1 as follows:

"The number of dynamic dose-responsive genes. The DDR is the number of dynamic dose-responsive genes. Linear, Poly2 and Poly3 are the numbers of DDR genes that are fitted by a linear, 2nd and 3rd order polynomial function respectively."

p.7 Caption of Figure 3 , it refers several times to figure numbers starting with 2. I suggest to include the sub-figure numbers (3A, 3B,….) next to each sub-figure.

We thank the reviewer, we corrected the figure numbers and we added the sub-figure letters

p.8 Caption of Figure 4. Similar to previous comment on Figure 3.

We thank the reviewer, we corrected the figure numbers and we added the sub-figure letters

Close
