# Supplementary material for: TinderMIX: Time-dose integrated modelling of toxicogenomics data
Source: Gigascience. 2020 May 25;9(5):giaa055. doi: 10.1093/gigascience/giaa055 (PMC7247400; doi:10.1093/gigascience/giaa055)
Supplement: giaa055_Response_to_Reviewer_Comments_Revision_1 [file giaa055_response_to_reviewer_comments_revision_1.pdf]

Author's Response To Reviewer Comments

Close

Manuscript ID: GIGA-D-20-0005

Title: Time-Dose INtegrated moDelling of toxicogenomics data

Dear editor,

please find enclosed a revised version of our manuscript. We addressed the comment raised by the reviewer.

We also registered the tool on the SciCrunch.org website and we obtained the RRID that we added into the “availability of source code and requirements” section.

We registered our tool on the GigaDB database, we obtained the DOI, and we cited the dataset in the manuscript in the “Availability of supporting data and materials” as you suggested.

We look forward to your positive feedback.

Yours Sincerely,
Dario Greco
PhD, Associate Professor,
Faculty of Medicine and Health Technology
Tampere University Finland

Reviewer 1
The authors have addressed my comments sufficiently.
I have only one minor comment:
Captions Additional file 2 and Additional file 3: adj.pval: pvalue of the fittied polynomial model -> adjusted p-value of the fitted polynomial model.

We have amended the captions as suggested by the reviewer.

Close
