# Supplementary material for: TinderMIX: Time-dose integrated modelling of toxicogenomics data
Source: Gigascience. 2020 May 25;9(5):giaa055. doi: 10.1093/gigascience/giaa055 (PMC7247400; doi:10.1093/gigascience/giaa055)
Supplement: giaa055_Reviewer_1_Report_Original_Submission — Diana Hendrickx -- 3/5/2020 Reviewed [file giaa055_reviewer_1_report_original_submission.pdf]

Reviewer Report

Title: TinderMIX: Time-Dose INtegrated moDelling of toxicogenomics data

Version: Original Submission Date: 3/5/2020

Reviewer name: Diana Hendrickx

Reviewer Comments to Author:

General :
In this paper, the authors propose a new approach, called TinderMIX, for simultaneously studying time and dose effects in toxicogenomics. Although many toxicogenomics experiments include data from different doses measured at different time points, only a few bioinformatics approaches to integrate time and dose effects exist, and their applications are limited to network identification and response modeling. The new method proposed by the authors extends existing methods by identifying genes with joined dose and time patterns of responsiveness, and predicting the molecular alteration values for the doses and time-points not included in the original experiment. Moreover, the approach assigns an activation label to each gene describing the time (early, middle, late) and dose effect (sensitive, intermediate, resilient). Genes are grouped based on the activation label, and pathway analysis can be performed on each group in order to better characterise the underlying biological mechanisms. Moreover, the method is available as a user-friendly R package on GitHub.
To the best of my knowledge, this is the first bioinformatics approach performing such an extensive analysis of both time and dose effects in toxicogenomics. This makes the method described in this manuscript of particular interest for improving our understanding of the mode of action of compounds. However, before I can recommend the manuscript for publication in GigaScience, a few issues have to be addressed:
Major :
* Materials and methods, section Time-dose Fitting: the authors explain that they use a desired activity threshold corresponding to a 10% increase/decrease with respect to controls. However, the authors have to explain why the threshold has been set to 10% and not to a higher or lower value. Moreover, the authors need to explain how the value of the threshold influences the results of their analysis. If the authors would have chosen a slightly lower or higher threshold, how much would their results differ from the current results?
* Caption Figure 3: "Considering the dose, the log-fold-change of sensitive-middle and sensitive-late genes mainly decreases as the dose increases." I agree with the authors that this is the case for the sensitive-late genes. However, for the sensitive-middle genes, I do not understand this conclusion. Looking at the figure for the sensitive-middle genes, I observe that the colored area in the right quadrants, displaying an increase in log-fold-change with increasing dose is larger than the area in the left quadrants, displaying a decrease. In particular, the purple bar is much larger than the red bar.
Minor :
* Abstract : The authors claim that 'Existing methods for toxicogenomics data analysis allow studying molecular alteration after the exposure (or treatment) at each time point individually.' Although this is

the case for the majority of the methods, there exist already a few methods for integrating the effects of time and dose, that consider the whole time profile instead of looking at each time point individually (see e.g. PMID: 31409281, PMID: 28032149, PMID: 31140561 (reference already mentioned by the authors in the introduction)). I therefore propose to replace "Existing methods …" by "The majority of existing methods …".

* Figure 1E: Explain symbols D+, D-, d+, d-, T+, T-, t+, t- in the figure caption, as you refer to the figure before explaining these symbols in the text.
* Materials and methods, section Enrichment of biological pathways: "KEGG enrichment analysis is performed by using the methodology implemented in the FunMappOne tool ??." Replace "??" by the reference to the paper on the FunMappOne tool.
* Materials and methods, section Dataset collection: "For every drug pathological score was computed by …" -> "Every drug pathological score was computed by …"
* Materials and methods, section Dataset collection: "Cyclosporine A and thioacetamide were selected as representative candidates for drugs with low and high toxic impact, respectively." Add literature references that explain that Cyclosporine A is a drug with low toxic impact and thioacetamide a drug with high toxic impact.
* Materials and methods, section Dataset collection: add link to website and literature reference for the Open TG-Gates dataset
* Materials and methods, section Dataset collection: "12 are replicates for each dose level". Are these biological or technical replicates? Please explain already in this paragraph that you use the in vivo data.
* Caption Table 1: "Linear is the number of DDR that is fitted by a liner, poly2 and poly3 are the numbers of DDR genes that are fitted by a linear, 2nd and 3rd order polynomial function respectively." -> "Linear, Poly2 and Poly3 are the numbers of DDR genes that are fitted by a linear, 2nd and 3rd order polynomial function respectively."
* Caption Figure 3 refers to Figures 2A - 2I. Has to be 3A-3I? Please label the figures with A, B, C, …, I. Also in the caption of Figure 3: "the effect of time is more evenly distributed across the possible combinations (Figure 2)." However, this information is not shown in Figure 2. Please refer to the correct figure.
* Additional files 2 and 3: I suggest to explain the abbreviations in the column names of the Excel tables in the description of the additional files.
* Caption Figure 4 refers to Figures 3A - 3I. Has to be 4A-4I? Please label the figures with A, B, C, …, I.
* Caption Figure 4: "direct correlation between the increase of the fold-change and the dose": authors refer to the purple and green bars. However, there are two types of green in the figures. Do you mean the blue green bars?
* The terms "Dynamic Doseâˆ'Responsive Increasing Area" and "Dynamic Doseâˆ'Responsive Decreasing Area" only appear in the caption of Figure 6. It would be more clear if these terms were already explained in the overview of the methods (Figure 1c + corresponding text).
* Conclusion: "pathways enrichment analysis" -> "pathway enrichment analysis".

Methods

Are the methods appropriate to the aims of the study, are they well described, and are necessary controls included? Choose an item.

Conclusions

Are the conclusions adequately supported by the data shown? Choose an item.

Reporting Standards

Does the manuscript adhere to the journal's guidelines on minimum standards of reporting? Choose an item.

Choose an item.

Statistics

Are you able to assess all statistics in the manuscript, including the appropriateness of statistical tests used? Choose an item.

Quality of Written English

Please indicate the quality of language in the manuscript: Choose an item.

Declaration of Competing Interests

Please complete a declaration of competing interests, considering the following questions:

- Have you in the past five years received reimbursements, fees, funding, or salary from an organisation that may in any way gain or lose financially from the publication of this manuscript, either now or in the future?
- Do you hold any stocks or shares in an organisation that may in any way gain or lose financially from the publication of this manuscript, either now or in the future?
- Do you hold or are you currently applying for any patents relating to the content of the manuscript?
- Have you received reimbursements, fees, funding, or salary from an organization that holds or has applied for patents relating to the content of the manuscript?
- Do you have any other financial competing interests?
- Do you have any non-financial competing interests in relation to this paper?

If you can answer no to all of the above, write 'I declare that I have no competing interests' below. If your reply is yes to any, please give details below.

I declare that I have no competing interests.

I agree to the open peer review policy of the journal. I understand that my name will be included on my report to the authors and, if the manuscript is accepted for publication, my named report including any attachments I upload will be posted on the website along with the authors' responses. I agree for my report to be made available under an Open Access Creative Commons CC-BY license (http://creativecommons.org/licenses/by/4.0/). I understand that any comments which I do not wish to be included in my named report can be included as confidential comments to the editors, which will not be published.

Choose an item.

To further support our reviewers, we have joined with Publons, where you can gain additional credit to further highlight your hard work (see: https://publons.com/journal/530/gigascience). On publication of this paper, your review will be automatically added to Publons, you can then choose whether or not to claim your Publons credit. I understand this statement.

Yes Choose an item.
