# Supplementary material for: TinderMIX: Time-dose integrated modelling of toxicogenomics data
Source: Gigascience. 2020 May 25;9(5):giaa055. doi: 10.1093/gigascience/giaa055 (PMC7247400; doi:10.1093/gigascience/giaa055)
Supplement: giaa055_Reviewer_2_Report_Original_Submission — Haralambos Sarimveis -- 3/30/2020 Reviewed [file giaa055_reviewer_2_report_original_submission.pdf]

Reviewer Report

Title: TinderMIX: Time-Dose INtegrated moDelling of toxicogenomics data

Version: Original Submission **Date:** 3/30/2020

Reviewer name: Haralambos Sarimveis

Reviewer Comments to Author:

The paper introduces a new toxicogenomics data modelling approach which considers simultaneously the effect of both time and dose in the calculation of points of departure. The method is applied on individual genes, but using enrichment analysis it is generalized and extended to the level of pathways and mechanisms of action.
The proposed method is novel, very interesting to the readers of GigaScience and paves the way for new developments in the area of chemicals safety and risk assessment by considering dose and time simultaneously. Therefore, I am happy to recommend the acceptance of the paper.
I have some minor comments that may contribute to further improve the manuscript and the presentation of the methodology:
1) The selected linear regression models include 1st , 2nd and 3rd order polynomials. These are not the usual models used in the standard BMD method (Gamma, Logistic models etc.). Is there any particular reason for this choice?
2) It is mentioned in p. 3 that the desired activity threshold is a 10% increase or decrease with respect to controls. How this is compared with Fig. 1 where the threshold seems to be higher than 10%?
3) What should be the minimum percentage of a region in the 3x3 grid belonging to the green (or red) area that defines it as a POD? For example, if a very small part of the sensitive-early region was green, it is still enough to specify it as a POD?
4) On the POD selection again, could there be a nonlinear case where the early-sensitive region is completely blue, but the early-intermediate and middle-sensitive are green? In this case, which region is selected as the POD?
Some typos:
Figure 1 caption: dow -> down
p. 6 Two question marks under "Enrichment of biological pathways"
p. 7 Caption of Table 1 is not syntactically correct.
p.7 Caption of Figure 3 , it refers several times to figure numbers starting with 2. I suggest to include the sub-figure numbers (3A, 3B,….) next to each sub-figure.
p.8 Caption of Figure 4. Similar to previous comment on Figure 3.

Methods

Are the methods appropriate to the aims of the study, are they well described, and are necessary controls included? Choose an item.

Conclusions

Are the conclusions adequately supported by the data shown? Choose an item.

Reporting Standards

Does the manuscript adhere to the journal's guidelines on minimum standards of reporting? Choose an item.

Choose an item.

Statistics

Are you able to assess all statistics in the manuscript, including the appropriateness of statistical tests used? Choose an item.

Quality of Written English

Please indicate the quality of language in the manuscript: Choose an item.

Declaration of Competing Interests

Please complete a declaration of competing interests, considering the following questions:

- Have you in the past five years received reimbursements, fees, funding, or salary from an organisation that may in any way gain or lose financially from the publication of this manuscript, either now or in the future?
- Do you hold any stocks or shares in an organisation that may in any way gain or lose financially from the publication of this manuscript, either now or in the future?
- Do you hold or are you currently applying for any patents relating to the content of the manuscript?
- Have you received reimbursements, fees, funding, or salary from an organization that holds or has applied for patents relating to the content of the manuscript?
- Do you have any other financial competing interests?
- Do you have any non-financial competing interests in relation to this paper?

If you can answer no to all of the above, write 'I declare that I have no competing interests' below. If your reply is yes to any, please give details below.

I declare that I have no competing interests

I agree to the open peer review policy of the journal. I understand that my name will be included on my report to the authors and, if the manuscript is accepted for publication, my named report including any attachments I upload will be posted on the website along with the authors' responses. I agree for my

report to be made available under an Open Access Creative Commons CC-BY license (http://creativecommons.org/licenses/by/4.0/). I understand that any comments which I do not wish to be included in my named report can be included as confidential comments to the editors, which will not be published.

Choose an item.

To further support our reviewers, we have joined with Publons, where you can gain additional credit to further highlight your hard work (see: https://publons.com/journal/530/gigascience). On publication of this paper, your review will be automatically added to Publons, you can then choose whether or not to claim your Publons credit. I understand this statement.

Yes Choose an item.
